# Structural basis of rotavirus RNA chaperone displacement and RNA annealing

**DOI:** 10.1073/pnas.2100198118

**Published:** 2021-10-06

**Authors:** Jack P. K. Bravo, Kira Bartnik, Luca Venditti, Julia Acker, Emma H. Gail, Alice Colyer, Chen Davidovich, Don C. Lamb, Roman Tuma, Antonio N. Calabrese, Alexander Borodavka

**Affiliations:** ^a^Department of Biochemistry, University of Cambridge, Cambridge CB2 1QW, United Kingdom;; ^b^Astbury Centre for Structural Molecular Biology, School of Molecular and Cellular Biology, Faculty of Biological Sciences, University of Leeds, LS2 9JT Leeds, United Kingdom;; ^c^Department of Chemistry, Center for NanoScience, Nanosystems Initiative Munich, Centre for Integrated Protein Science Munich, Ludwig Maximilian University of Munich, D-81377 Munich, Germany;; ^d^Department of Biochemistry and Molecular Biology, Biomedicine Discovery Institute, Faculty of Medicine, Nursing and Health Sciences, Monash University, Clayton, VIC 3800, Australia;; ^e^Australian Research Council (ARC) Centre of Excellence in Advanced Molecular Imaging, European Molecular Biology Laboratory (EMBL) Australia, Clayton, VIC 3800, Australia;; ^f^Faculty of Science, University of South Bohemia, 370 05 Ceske Budejovice, Czech Republic

**Keywords:** RNA chaperones, ribonucleoproteins, genome assembly, rotavirus

## Abstract

Rotavirus genomes are distributed between 11 distinct RNA molecules, all of which must be selectively copackaged during virus assembly. This likely occurs through sequence-specific RNA interactions facilitated by the RNA chaperone NSP2. Here, we report that NSP2 autoregulates its chaperone activity through its C-terminal region (CTR) that promotes RNA–RNA interactions by limiting its helix-unwinding activity. Unexpectedly, structural proteomics data revealed that the CTR does not directly interact with RNA, while accelerating RNA release from NSP2. Cryo–electron microscopy reconstructions of an NSP2–RNA complex reveal a highly conserved acidic patch on the CTR, which is poised toward the bound RNA. Virus replication was abrogated by charge-disrupting mutations within the acidic patch but completely restored by charge-preserving mutations. Mechanistic similarities between NSP2 and the unrelated bacterial RNA chaperone Hfq suggest that accelerating RNA dissociation while promoting intermolecular RNA interactions may be a widespread strategy of RNA chaperone recycling.

## Significance

Accurate RNA folding is essential for virus replication. Rotaviruses are viruses infecting humans and animals. Rotavirus genome comprises 11 distinct RNAs, and successful replication requires the incorporation of all 11 RNAs into a virion. The RNA chaperone NSP2 binds viral transcripts, regulating their interactions with each other. NSP2 must release RNAs after they base pair prior to their packaging. Using single-molecule fluorescence tools, we dissected the individual steps of the RNA chaperone activity of NSP2. Structural proteomics and cryo-EM studies of the NSP2–RNA complex revealed that NSP2 regulates RNA unfolding and the release of the RNA using its charged C-terminal region. Some aspects of the viral RNA chaperone regulation mirror the conserved autoregulation mechanisms employed by bacterial RNA chaperones.

Selective incorporation of viral genomes into nascent virions is essential for virus replication. This process is highly challenging for RNA viruses with multisegmented genomes (including rotaviruses [RV]) since they must coordinate the selection and assembly of multiple distinct RNAs (1, 2). Despite these challenges, RVs achieve highly efficient, selective, and stoichiometric assembly of their 11 genome segments through a series of redundant, sequence-specific, intermolecular RNA–RNA contacts (3). While RNA–RNA interactions may underpin genome assembly, the remarkable selectivity of these interactions is determined by a complex network of RNA–protein interactions (4). In RVs, the viral RNA chaperone protein NSP2 facilitates sequence-specific, intersegment RNA–RNA interactions to ensure robust assembly of complete viral genomes (5, 6).

NSP2 is a multivalent, nonspecific RNA chaperone with high nanomolar affinity for single-stranded (ss) RNA (4, 7, 8). This allows it to both act as a matchmaker of intermolecular duplexes and limit transient, nonspecific RNA–RNA interactions (4, 9). This creates a mechanistic conundrum, as NSP2 has to balance helix unwinding and RNA annealing in order to achieve accurate and stoichiometric assembly of distinct RNAs. As such, this viral RNA chaperone plays an absolutely critical role in RV replication (10, 11).

**Fig. 1.**
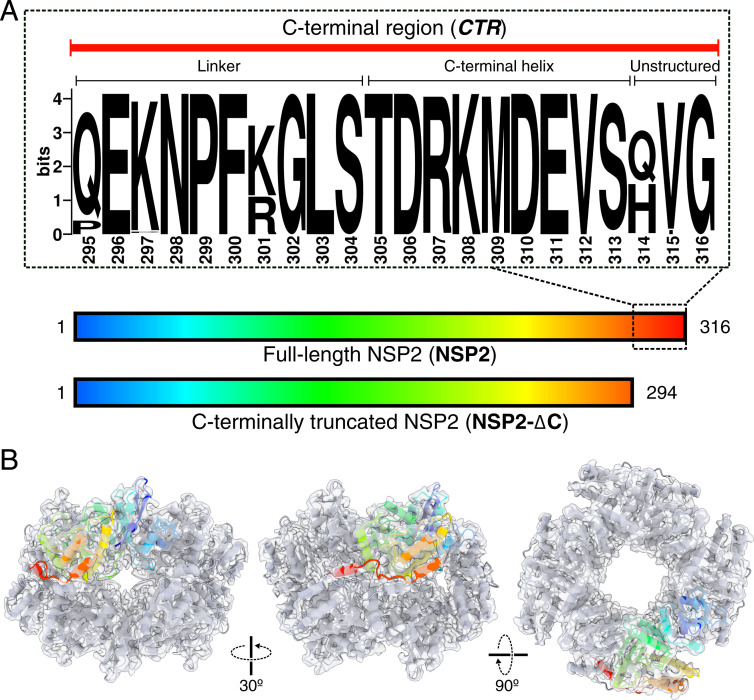
Structure and conservation of NSP2 CTR. (*A*) Constructs of full-length NSP2 (NSP2) and NSP2-∆C (residues 1 through 294) used in this study. An expanded, annotated sequence logo of the NSP2 CTR is shown, which consists of an unstructured, flexible linker region (residues 295 through 304) and a single alpha helix (CTH, residues 305 through 313). Downstream residues (314 through 316) are nonessential for viral replication. Sequence conservation and multiple sequence alignment were performed using full-length NSP2-coding sequences of group A RVs, as described in *Materials and Methods*. (*B*) A 3.9-Å resolution cryo-EM reconstruction of the octameric NSP2 apoprotein (gray transparent surface) with associated model (cartoon). A single monomer is highlighted and color-coded according to sequence position shown in *A*.

Previous mutational studies of NSP2 have been hindered by the lack of a robust reverse-genetics system (12–15). As such, the only region of NSP2 experimentally demonstrated as essential for virus replication to date is the C-terminal region (CTR) (residues 295 through 316) (Fig. 1*A*) (16–18). We have recently demonstrated that C-terminally truncated NSP2 (NSP2-∆C) has significantly reduced RNA annealing activity in vitro (19). This collectively suggests that the CTR is required for the RNA chaperone activity of NSP2, although its exact functional role(s) remained unclear.

Here, we have used a single-molecule fluorescence spectroscopy approach to decouple the RNA annealing and RNA unwinding activities of full-length NSP2 and NSP2-∆C. While NSP2-∆C exhibits a reduced capacity to promote RNA–RNA interactions, it possesses enhanced RNA unwinding activity. To resolve these paradoxical observations, we determined cryogenic electron microscopy (cryo-EM) structures of NSP2 and an NSP2–ribonucleoprotein (RNP) complex at global resolutions of 3.9 Å and 3.1 Å, respectively. In the RNP structure, the RNA density localized to surface-exposed, positively charged grooves, with no evidence of the CTR interacting with the RNA. To directly map the RNA-binding surfaces of NSP2, we employed complementary structural proteomics tools that revealed that all RNA–protein contacts required for nonspecific, high-affinity RNA recognition were outside the CTR.

Furthermore, our data show that while the CTR does not directly interact with RNA, it contains a conserved acidic patch that is poised toward bound RNA. Mechanistically, we demonstrate that that the CTR promotes RNA release, indicating that the CTR is required for preventing the formation of a highly stable, kinetically trapped RNP complex that is not conducive to RNA–RNA annealing. To validate our model, we showed that alanine substitutions of conserved acidic residues in the CTR (D306, D310, and E311) abrogated RV replication, while charge-preserving mutations had no detrimental effect on virus rescue in reverse genetics experiments. Our multifaceted approach provides a mechanistic basis for RNA release from high-affinity capture by NSP2, which is required for RNA annealing, chaperone dissociation, and ultimately efficient selection and packaging of a complete RV genome.

## Results

### Conserved NSP2 CTR Is Required for Efficient RNA Annealing.

The CTR of NSP2 consists of a flexible linker (residues 295 through 304) that tethers an α-helix (C-terminal helix, CTH) to NSP2_core_ (i.e., residues 1 through 294, Fig. 1*A*). The CTH is ampholytic, containing highly conserved, positively (Arg307, Lys308) and negatively charged (Asp306, Asp310, and Glu311) residues. To interrogate the role of the CTR in NSP2 function, we generated NSP2-∆C (residues 1 through 294) lacking the entire CTR (*SI Appendix*, Fig. S1). This NSP2-∆C construct has been previously characterized by others (10, 20) (Fig. 1).

To visualize the CTR conformation in solution, we determined a cryo-EM three-dimensional (3D) reconstruction of full-length NSP2 (henceforth referred to as NSP2) at a global resolution of 3.9 Å (Fig. 1*B* and *SI Appendix*, Figs. S2 and S3). As expected, our cryo-EM-derived model of NSP2 revealed an octameric assembly, which was highly similar to previously solved crystal structures of NSP2 (the overall RMSD between equivalent Cα atoms of the refined model presented here and PDB 1L9V is 1.124 Å) (7, 10, 20). Within our density map, the CTH exhibited well-resolved density (local resolutions ranging between 3.6 and 4.0 Å (*SI Appendix*, Fig. S2). Due to intrinsic flexibility, the linker region was poorly resolved (*SI Appendix*, Fig. S3). We also determined a 3D reconstruction of NSP2-∆C using negative-stain EM which confirmed that NSP2-∆C remains octameric, demonstrating that the CTR does not play a role in the assembly of NSP2 into functional octamers (*SI Appendix*, Fig. S1).

**Fig. 2.**
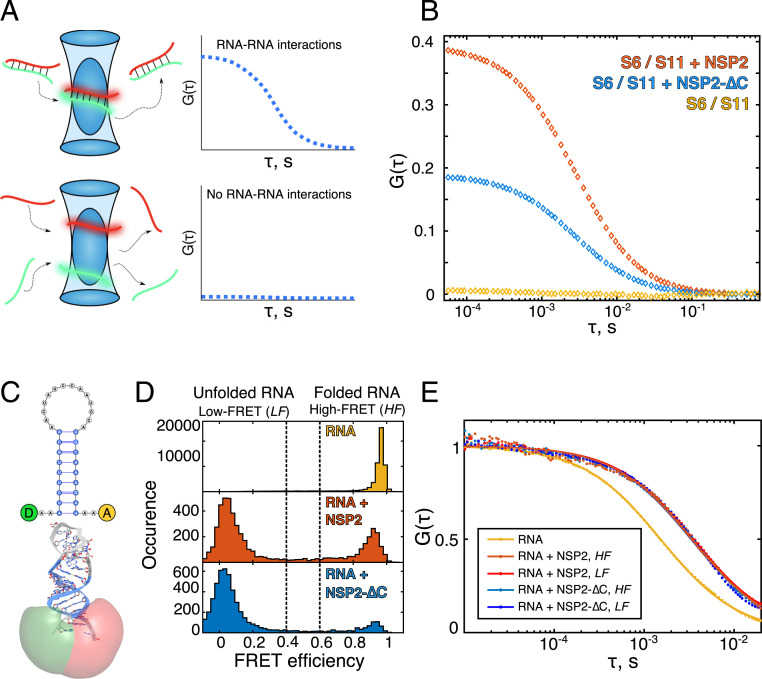
The NSP2 CTR is required for efficient RNA–RNA matchmaking yet limits RNA unwinding. (*A*) Single-molecule assays to probe RNA–RNA interactions between partially complementary fluorescently labeled RNAs S6 (green) and S11 (red). Upon strand annealing, differently labeled transcripts codiffuse (shown as a duplex within the blue confocal volume). Such interactions result in a nonzero amplitude of the CCF and thus directly report the fraction of interacting RNAs. A CCF amplitude G(τ) = 0 indicates that the two RNA molecules diffuse independently and are not interacting. (*B*) An equimolar mix of S6 and S11 RNAs were coincubated in the absence (yellow) or presence of either NSP2 (orange) or NSP2-∆C (blue). While the two RNAs do not interact, coincubation with NSP2 results in a high fraction of stable S6:S11 complexes. In contrast, coincubation of S6 and S11 with NSP2-∆C results in twofold reduction of the fraction of S6:S11 complexes. (*C*) Schematics of the RNA stem-loop used for the smFRET studies of helix-unwinding activity. The FRET donor (D, green) and acceptor (A, red) dye reporters (Atto532 and Atto647N) and their calculated accessible volumes (green and red, respectively) are shown. (*D*) smFRET efficiency histograms of the RNA stem-loop alone (*Top*, yellow) in the presence of 5 nM NSP2 (*Middle*, red) or 5 nM NSP2-∆C (*Bottom*, blue). (*E*) A species-selective correlation analysis was performed on the high FRET (*HF*) and low FRET (*LF*) species of RNA stem-loops bound to NSP2 (orange) and NSP2-∆C (blue). All FCS analyses were performed on the smFRET data shown in *D*.

Next, we investigated the role of the CTR in the RNA annealing activity of NSP2 using a fluorescence cross-correlation based RNA–RNA interaction assay (19). We chose RNA transcripts S6 and S11, representing RV gene segments 6 and 11, as these have been previously shown to form stable RNA–RNA contacts in the presence of NSP2 (19). In brief, fluorescently labeled transcripts (RNAs S6 and S11) were coincubated in the absence or presence of either NSP2 or NSP2-∆C, and intermolecular interactions were then quantitated in solution by measuring the cross-correlation function (CCF) amplitudes (Fig. 2*A*). While a zero CCF amplitude is indicative of noninteracting RNAs, increasing yields of intermolecular interactions result in proportionally higher, nonzero CCF amplitudes (21).

Coincubation of S6 and S11 transcripts alone resulted in a near-zero CCF amplitude indicating that they do not spontaneously interact (Fig. 2*B* and *SI Appendix*, Fig. S3). In contrast, addition of NSP2 to an equimolar mixture of these two RNAs produced a high CCF amplitude, indicative of intermolecular RNA duplex formation (Fig. 2*B*). This observation is in agreement with the known role of NSP2 as an RNA chaperone, facilitating the remodelling and annealing of structured RNAs (19).

Coincubation of S6 and S11 in the presence of NSP2-∆C resulted in a reduced CCF amplitude (Fig. 2*B*), indicating that NSP2-∆C has a reduced RNA-annealing activity relative to full-length NSP2. This is in agreement with our previous observation that NSP2-∆C has reduced capacity to promote interactions between RV RNAs (19). Our combined data confirms that the CTR plays a role in the RNA chaperone function of NSP2 irrespective of the RNA substrates chosen.

### The NSP2 CTR Reduces the RNA-Unwinding Activity but Does Not Directly Interact with RNA.

As the ability of NSP2 to unfold and remodel RNA structures is a prerequisite for its RNA-annealing activity (4), we next investigated the role of the CTR in RNA helix destabilization. We used single-molecule Förster Resonance Energy Transfer (smFRET) to directly compare the abilities of NSP2 and NSP2-∆C to unwind an RNA stem-loop labeled at the 5′ and 3′ termini with donor and acceptor dyes (Atto532 and Atto647N) (Fig. 2*C* and *SI Appendix*, Fig. S4).

In the absence of either protein, the stem-loop alone adopts a folded conformation, resulting in a single, high-FRET population (*E*_FRET_ = ∼0.95) (Fig. 2*D*). Incubation with NSP2 produces two distinct FRET populations, corresponding to fully folded (*E*_FRET_ = ∼0.95) and unfolded (*E*_FRET_ = ∼0.05) RNA states. No intermediate FRET populations (corresponding to partially unwound stem-loop conformations) were observed, in agreement with previous observations of NSP2-mediated RNA-unwinding (4).

We then measured the ability of NSP2-∆C to unwind this RNA stem-loop. Surprisingly, in the presence of NSP2-∆C, the stem-loop was predominantly unfolded (*E*_FRET_ = ∼0.05) (Fig. 2*D*). Furthermore, we did not observe differences in binding of either NSP2 or NSP2-∆C to both folded and unfolded RNA conformations (Fig. 2*E*). These data demonstrate that NSP2-∆C has enhanced RNA unfolding activity compared to its full-length counterpart. This result is somewhat paradoxical: while NSP2-∆C is more efficient at destabilizing RNA structure (Fig. 2*D*), it is approximately half as efficient at promoting the annealing of structured RNAs as NSP2 (Fig. 2*B*).

**Fig. 3.**
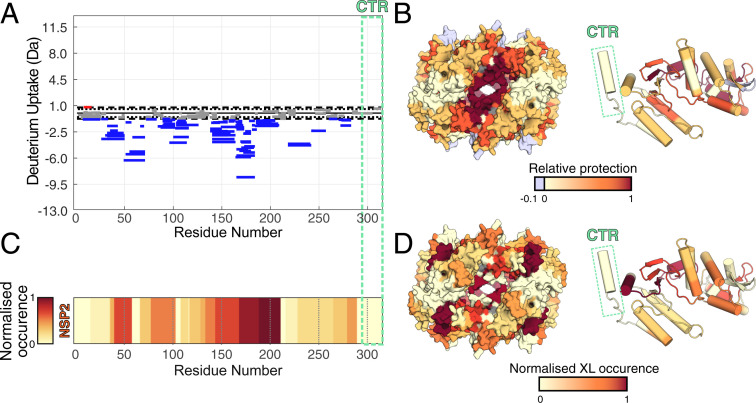
The CTR does not interact with RNA. (*A*) Differences in the deuterium uptake in NSP2 (integrated over four different HDX timepoints), for NSP2 alone and NSP2–RNA complex. Protected and deprotected peptides are colored blue and red, respectively. Peptides with no significant difference between conditions, determined at a 99% CI (dotted line), are shown in gray. Green dashed box corresponds to the CTR, revealing no significant differences in deuterium exchange in the presence or absence of RNA. (*B*) A differential HDX map colored onto the NSP2 octamer surface (*Left*) and monomer structure (*Right*). Multiple regions of NSP2 except the CTR (green box) are protected by bound RNA. (*C*) Normalized occurrence of the RNA-interacting peptides determined using UV crosslinking (identified by RBDmap). (*D*) RBDmap-identified RNA-binding peptides mapped onto the surface of NSP2 octamer (*Left*) and its monomer (*Right*). Structures are colored according to frequency of crosslink occurrence. No RNA:peptide cross-links were identified on the CTR (green box).

To deduce whether the CTR directly interacts with RNA, we used a combination of structural proteomics techniques (Fig. 3). We performed hydrogen–deuterium exchange-mass spectrometry (HDX-MS) experiments to map regions of NSP2 that become protected from deuterium exchange in the presence of RNA, presumably as they are involved in RNA binding and occluded from solvent when bound. We observed significant protection from exchange for peptides that predominantly mapped to ∼25 Å-deep grooves present on the surface of NSP2, indicating that this is the major RNA-binding site of NSP2 (Fig. 3*A*). Intriguingly, we did not observe any significant change in protection for peptides that spanned the CTR, indicating that the CTR does not directly interact with RNA (Fig. 3 *A* and *B* and *SI Appendix*, Fig. S5).

We further corroborated the location of RNA-binding sites on NSP2 using UV crosslinking with RBDmap (22, 23). In addition to HDX-MS data, RBDmap identified RNA-linked peptides that map to these surface-exposed RNA-binding grooves (Fig. 3 *C* and *D* and *SI Appendix*, Fig. S5). However, we again did not observe any RNA-linked peptides corresponding to the CTR.

Collectively, these results reinforce the notion that the CTR is involved in the RNA chaperone activities of NSP2. Our data indicates that although the CTR does not directly interact with RNA, it is a determinant of both the RNA-unwinding and -annealing activities of NSP2.

### Cryo-EM Visualization of NSP2–RNA Interactions.

**Fig. 4.**
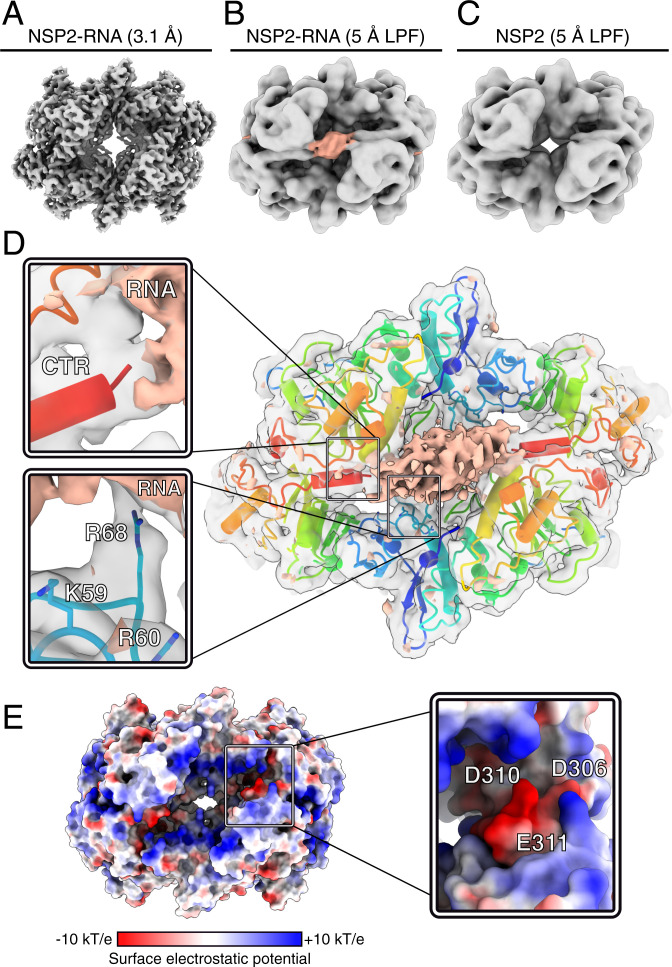
Cryo-EM structure of the NSP2–RNP complex. (*A*) A 3.1-Å-resolution reconstruction of the NSP2–RNP complex. (*B* and *C*) Cryo-EM maps of NSP2–RNA (*B*) and NSP2 apoprotein (*C*) LPF to 5 Å. A cryo-EM density feature (peach) is attributed to bound RNA in the LPF RNP map. Both maps are reconstructed with D4 symmetry. (*D*) Direct visualization of interactions between NSP2 and RNA using C4 symmetry expansion and focused classification. The positive difference density map corresponding to RNA (peach) is overlaid onto the unsharpened NSP2–RNP complex map determined through symmetry expansion and focused classification (gray, transparent density) and atomic model of NSP2. (*Inset*) Zoom-in of the CTR positioned relative to RNA density (*Top*) and RNA-interacting residues (*Bottom*). (*E*) The surface electrostatic potential analysis of NSP2. (*Inset*) Zoom-in of the CTR, with residues within the acidic patch (D306, D310, and E311) annotated.

To understand the molecular basis of RNA binding by NSP2, we determined a cryo-EM reconstruction of an NSP2–RNP complex at a global resolution of 3.1 Å (Fig. 4*A* and *SI Appendix*, Figs. S1 and S2). While the cryo-EM density corresponding to NSP2 was well resolved, there was no density that could be attributed to the RNA in the high-resolution postprocessed NSP2–RNP map (Fig. 4*A*). This is likely due to the heterogeneity and intrinsic flexibility of NSP2-bound unstructured single-stranded RNA. Despite this, an additional feature localized to RNA binding sites identified by HDX-MS and RBDmap (Fig. 3) was present in 5-Å low-pass filtered (LPF) maps (Fig. 4*B*). Notably, such a density feature was not present in 5-Å LPF NSP2 apoprotein maps (Fig. 4*C*). We attribute this density to NSP2-bound RNA.

To improve visualization of the RNA density, we performed focused classification using C4 symmetry-expanded data with a mask applied to a single RNA-binding face of NSP2 (24–26). The resulting 3D reconstructions readily classified into four dominant populations, three of which had poor RNA occupancy (each class with 26% of the input particles), while a single 3D class average (22% of input particles) exhibited improved RNA density (*SI Appendix*, Fig. S2). Due to the reasons outlined above, the diffuse nature of this density prevented us from modeling the ssRNA into the structure. However, we were able to visualize residue-specific NSP2–RNA contacts (Fig. 4*D*).

We built an atomic model of NSP2 into the sharpened map and then computed a difference map between NSP2 and the RNA-occupied, focused map in order to visualize NSP2–RNA contacts. Significant positive density was localized in the basic groove of NSP2 (Fig. 4), consistent with the binding site identified through HDX and RBDmap (Fig. 3). We observed interactions between positively charged residues, most notably R68 (Fig. 4*D*). Adjacent to this contact are K58, K59, and R60, of which K59 and R60 are directly oriented toward the RNA density (Fig. 4 *D*, *Inset*). The importance of these residues for RNA capture by NSP2 is strongly supported by previous biochemical studies that identified a number of solvent-exposed lysine and arginine residues (K37, K38, K58, K59, R60, and R68) that span the periphery of the NSP2 octamer (*SI Appendix*, Fig. S6) and contribute to RNA binding (27).

Furthermore, the identified residues are localized to an unstructured loop within the RNA-binding groove, allowing promiscuous and flexible accommodation of alternative RNA structures with near-identical affinities by NSP2, consistent with previous reports (4, 28). Together with our HDX and RBDmap results, our cryo-EM reconstruction reveals a number of electrostatic contacts that provide a plausible molecular basis for nonspecific NSP2–RNA interactions (Fig. 4 *D*, *Inset*). In addition, our EM reconstruction has revealed a number of other residues (R240, K286, and F290) that likely contribute to RNA binding, also identified by HDX and RBDmap (*SI Appendix*, Figs. S5 and S6). These residues may also participate in nonspecific RNA contacts via electrostatic interactions, hydrogen bonding, and π-π stacking, consistent with a significant nonelectrostatic contribution to the overall free energy of RNA binding to NSP2.

### Conserved Acidic Patch within the CTR Promotes RNA Dissociation.

**Fig. 5.**
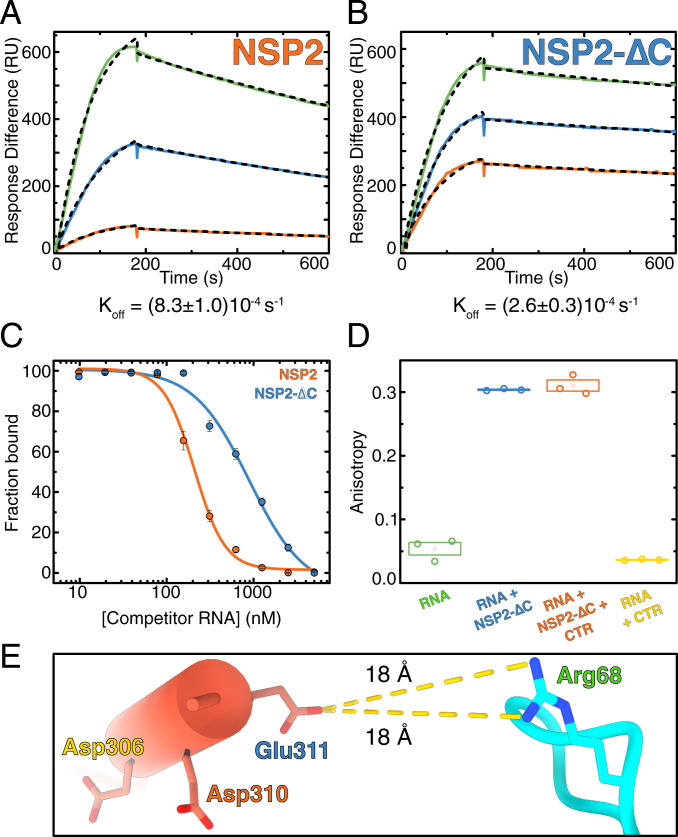
The CTR promotes RNA dissociation noncompetitively. (*A* and *B*) SPR sensograms of NSP2 (*A*) and NSP2-∆C (*B*) binding to RNA. Although NSP2-∆C binds RNA with an approximately sixfold higher affinity, this is due to a modest (1.5-fold) increase in *K*_on_ and a larger (3.2-fold) decrease in *K*_off_. (*C*) RNA competition assay. The fractional binding of fluorescently labeled RNA was determined by fluorescence anisotropy. Labeled RNA (10 nM) fully bound to NSP2 (orange) or NSP2-∆C (blue) was titrated with unlabeled RNA of identical sequence to compete for NSP2 binding against labeled RNA. The IC_50_ values for NSP2 and NSP2-∆C are 208 ± 11 nM and 890 ± 160 nM, respectively. The NSP2–RNA complex undergoes strand exchange more readily than the NSP2–∆C:RNA complex. (*D*) RNA binding by NSP2 in the presence of the CTR peptide. CTR peptide (10 µM) was added to preformed NSP2–∆C:RNA complexes. The CTR peptide does not compete with RNA for binding to NSP2-∆C. (*E*) Estimated distances between acidic residues within the CTR and the R68 that interacts with RNA. Note the nearest side chain (E311), which is 18 Å away from R68.

Within the cryo-EM density map, the CTRs are poised below the RNA, while making limited contacts with the observed RNA density (Fig. 4*D*). This suggests that the CTR may play a role in promoting RNA dissociation from NSP2. To investigate this, we performed binding kinetics measurements using surface plasmon resonance (SPR) (Fig. 5 *A* and *B*). Association rate constants (*K*_on_) remain largely consistent across a range of concentrations of both NSP2 and NSP2-∆C (NSP2-∆C binds (1.5 ± 0.4)-fold faster than NSP2) (*SI Appendix*, Table S1). However, NSP2-∆C exhibited a (3.2 ± 0.3)-fold slower dissociation rate than NSP2, suggesting a role for the CTR in the displacement of bound RNA (Fig. 5*A* and *SI Appendix*, Table S1).

Close examination of our cryo-EM-derived model revealed a conserved acidic patch within the CTR (Fig. 4*E*). This is in contrast to other clusters of surface-exposed acidic residues on NSP2 that show a low degree of conservation (*SI Appendix*, Fig. S6). The acidic patch of the CTR is presented directly underneath the density attributed to bound RNA (Fig. 4*D*), potentially promoting RNA displacement from NSP2. Such displacement could be achieved either via direct competition with RNA-binding residues or by providing a negatively charged environment that accelerates RNA dissociation from NSP2 through charge repulsion.

Therefore, to further investigate RNA displacement from NSP2, we used RNA competition assays (Fig. 5 *C* and *D*). We performed titrations of unlabeled RNA into preformed RNP complexes containing fluorescently labeled RNA to understand the differences in RNA exchange and chaperone recycling between NSP2 and NSP2-∆C. Using fluorescence anisotropy, we estimated the degree of competition as the concentration of competitor RNA required to displace 50% of prebound RNA from either NSP2 or NSP2-∆C complexes (IC_50_). We determined IC_50_ values of 208 ± 11 nM and 890 ± 160 nM for NSP2 and NSP2-∆C, respectively, confirming that NSP2-∆C undergoes approximately fourfold reduced RNA exchange, consistent with its approximately threefold slower rate of dissociation from RNA (Fig. 5 *A* and *B*).

We then investigated whether the CTR promotes RNA dissociation from NSP2 through directly competing with RNA for binding to basic, RNA-binding residues on the NSP2_core_. To achieve this, we measured RNA binding by NSP2-∆C in the presence of saturating amounts of a synthetic peptide matching the sequence of the CTR. We confirmed that the peptide in isolation was structured and able to directly bind to NSP2-∆C octamers when added in trans (*SI Appendix*, Fig. S7). No dissociation of RNA from NSP2-∆C was observed in the presence of 20-fold molar excess of the CTR peptide over NSP2-∆C (Fig. 5*D*). Furthermore, no RNA binding was observed upon incubation with 10 µM CTR peptide (i.e., 400-fold excess), indicating that the CTR does not bind RNA. This suggests that while the CTR is required for RNA displacement from NSP2, this does not occur through direct competition. However, from these data we cannot definitively rule out a model whereby the CTR promotes dissociation in cis (i.e., as part of the intact NSP2 octamer) via increasing the effective concentration of the CTR in proximity to bound RNA in a displacement reaction.

We analyzed our atomic model of NSP2 to evaluate the distances between acidic residues within the CTR and the basic, RNA-binding residues localized to flexible loops within the RNA-binding grooves (Fig. 5*E* and *SI Appendix*, Fig. S6). The distances (∼10 to 30 Å) between acidic residues within the CTR and the RNA-interacting residues are incongruent with a direct competition model. While R68 was demonstrated to directly interact with RNA (Fig. 4*D*), it is 18 Å away from acidic residues within the CTR. This further demonstrates that while the CTR promotes dissociation of RNA from NSP2, it does not do so through direct competition for NSP2_core_ binding (Fig. 5*E*). Collectively, our data suggest that conserved acidic patches within the CTR promote dissociation of bound RNA from NSP2 via charge repulsion.

### CTR Acidic Patch Is Required for Viral Replication.

**Fig. 6.**
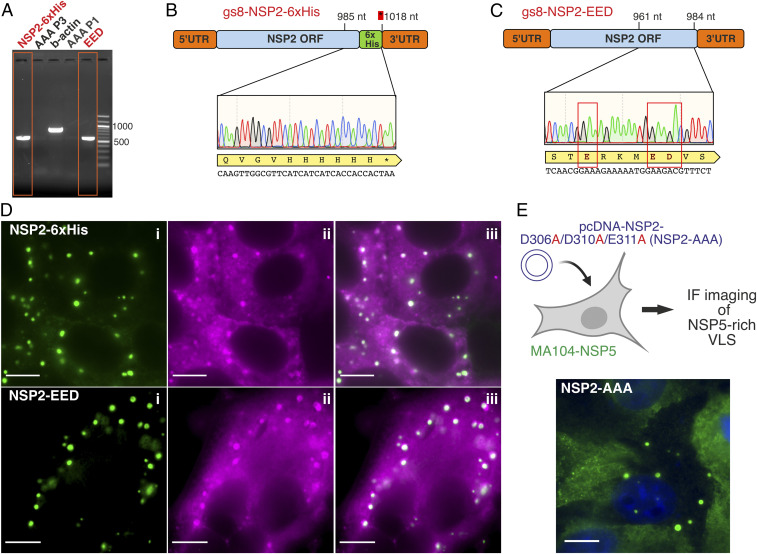
CTR acidic patch is essential for viral replication. (*A*) Rescue of recombinant RVs harboring mutated NSP2 sequences. RT-PCR results of the RNA extracted from MA104 cells infected with lysates from reverse genetics experiments (*Materials and Methods*) designed to rescue C-terminally 6× His-tagged NSP2 (“NSP2-6xHis”) and the charge-preserving mutant NSP2-EED, with NSP2-sequencing results shown in *B* and *C*, respectively. Attempts to rescue NSP2-AAA mutant were unsuccessful, with no viral RNA detected by RT-PCR after up to three blind passages (AAA-P1 and AAA-P3). For each experiment, three independent attempts were made to rescue the wild-type virus and the mutants. (*D*) NSP2 mutants form functional viral replication factories (viroplasms) in infected cells. After recombinant RV rescue, cell lysates were applied to NSP5-EGFP cells to monitor viroplasm formation at 6 h after infection, visualized by fluorescence microscopy (green inclusions, *Inset* i). smFISH revealed that these viroplasms contained viral RNAs (magenta, *Inset* ii, and panel iii show an overlay of both EGFP-NSP5 and RNA channels), confirming that they represent bona fide replication factories. (*E*) Nonreplicating NSP2-AAA mutant examined by coexpression with untagged NSP5 in a MA104-NSP5 cell line. Viroplasm-like structures were visualized by immunostaining of NSP5 (green). Dark blue: DAPI-stained nuclei. (Scale bars, 10 μm.)

To validate our findings, we employed a reverse-genetics approach to rescue recombinant RVs with point mutations within the CTR. We assessed the effects of amino acid substitutions within the CTR on viral replication by attempting recombinant virus rescue (*Materials and Methods*). Five attempts to rescue a triple-alanine mutant D306A/D310A/E311A (referred to as AAA) were unsuccessful (Fig. 6*A*), suggesting that these mutations abrogate virus replication. Remarkably, a triple mutant containing charge-preserving mutations D306E/D310E/E311D (referred to as EED) was successfully rescued along with the wild-type virus (Fig. 6*A* and *SI Appendix*, Fig. S8). This directly demonstrates the essential role of the CTR acidic patch in RV replication.

During the course of infection, RV assembly occurs in large, cytoplasmic viral replication factories, assembled from NSP2 and NSP5 and containing viral RNAs (2, 16, 29–31). While the C-terminal hexa-histidine–tagged NSP2 (NSP2-6xHis) and the EED mutant were able to support viral replication (Fig. 6 *A*–*C*), we sought to determine whether these mutants had any effect on replication factory assembly. Remarkably, neither C-terminal 6xHis-tagging of NSP2 nor introduction of the charge-preserving mutations had any noticeable impact on the formation of viroplasms (Fig. 6*D*) at 6 h postinfection in MA104 cells expressing NSP5-EGFP (Fig. 6*D*). Single-molecule (sm) fluorescence in situ hybridization (FISH) (*Materials and Methods*) confirmed that these NSP5-EGFP–tagged inclusions represented functional viroplasms that support accumulation of viral RNAs. Importantly, the kinetics of viroplasm formation (e.g., readily observable <6 hpi) were comparable to those observed for wild-type virus replicating in the NSP5-EGFP cell line (31, 32).

Since all our attempts to rescue NSP2-AAA mutants were unsuccessful, we also examined whether these mutations disrupted the formation of the NSP2/NSP5-rich viroplasms. As described in *Materials and Methods*, we transfected an NSP2–AAA-expressing DNA construct into MA104 cells stably expressing NSP5 (29, 30) and analyzed the formation of viroplasm-like structures through immunofluorescence imaging of NSP5 (Fig. 6*E*). We observed that NSP2-AAA supported the formation of NSP5/NSP2-rich cytoplasmic inclusions morphologically identical to those formed by wild-type virus (31), allowing us to conclude that NSP2-AAA mutant retained its capacity to form viroplasm-like structures. Together, these results further confirm that the conserved charged residues within the acidic patch of the NSP2 CTR are essential for virus replication.

## Discussion

Long RNAs adopt an ensemble of diverse stable structures that limit spontaneous RNA–RNA interactions through the sequestration of sequences required for intermolecular base pairing (19, 33–36). This necessitates the action of RNA chaperone proteins to bind and refold RNA structures in order to promote RNA-annealing between complementary sequences (37–39).

In order to function as an RNA chaperone, NSP2 must capture, unwind, anneal, and release complementary RNA sequences (19, 40, 41). Previous structural studies have provided static snapshots of crystallographically averaged NSP2–RNA complexes (7, 20, 27). However, due to the highly dynamic nature of the protein–RNA interactions required for its RNA chaperone activity, they have only revealed limited insights into the molecular mechanisms of NSP2. Our recent work (4, 19, 42) indicates that for NSP2–RNP complexes, such heterogeneity arises from poorly defined protein–RNA stoichiometries and the ability of bound RNA to adopt multiple configurations and orientations. To overcome these challenges, we used a combination of single-molecule fluorescence, cryo-EM, structural proteomics, and biophysical assays to decipher the mechanism of NSP2 chaperone function.

Previous work suggests that the CTR of NSP2 is essential for RV replication (17). Using single-molecule fluorescence techniques, we have directly shown that the CTR of NSP2 is important for promoting RNA–RNA interactions. However, we only identified interactions between RNA and basic residues located in flexible loops within the RNA-binding groove of NSP2 but not the CTR. Remarkably, similar RNA recognition mechanisms have been reported in other RNA chaperones including *Escherichia coli* StpA and HIV-1 NC (43, 44). Collectively, these results highlight the role of the CTR in NSP2 RNA chaperone activity but not RNA binding.

### Mechanism of the CTR-Assisted RNA Displacement and Its Role in RNA Matchmaking.

**Fig. 7.**
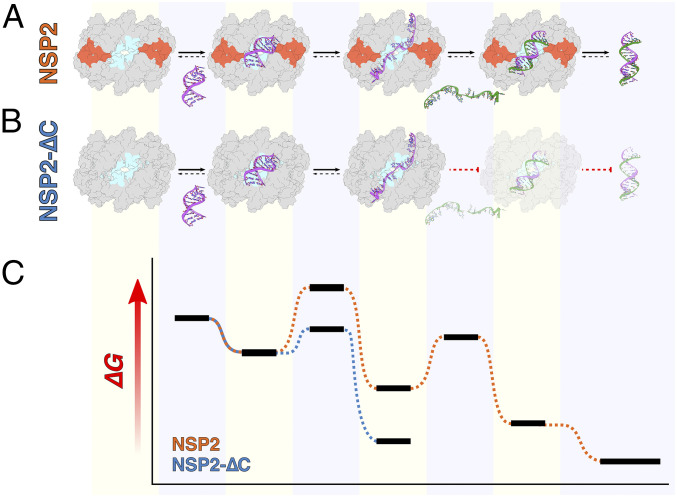
Proposed mechanism of CTR-accelerated RNA dissociation and its requirement for efficient NSP2-mediated RNA–RNA interactions. (*A*) NSP2 captures RNA (purple) via a positively charged groove (cyan) resulting in RNA unwinding. Binding of a second, complementary RNA strand (green) by NSP2 allows efficient annealing, and the proximity to the CTR (burnt orange) promotes dissociation of dsRNA from NSP2. (*B*) In contrast, NSP2-∆C captures and unwinds RNA, forming a highly stable intermediate. The stability of the intermediate state makes displacement of the bound RNA by a complementary RNA segment via annealing thermodynamically unfavorable. (*C*) A free energy diagram of NSP2 (orange) and NSP2-∆C (blue)-mediated RNA annealing. Horizontal black bars correspond to the free-energy levels of different RNA states corresponding to the above schematic representations in *A* and *B*.

We propose a model whereby the RV RNA chaperone NSP2 binds to RNA with high affinity, resulting in RNA structure destabilization (Fig. 7*A*). Fluorescence correlation spectroscopy (FCS) analysis of high- and low-FRET RNA species points out that full-length NSP2 binds to both the unfolded and folded RNA conformations, priming RNAs for efficient RNA annealing (Fig. 2). These results are fully consistent with our previous single-molecule fluorescence studies of NSP2 (4), confirming that upon binding to NSP2, only two FRET states are observed—a high-FRET state corresponding to a folded RNA stem-loop and a low FRET state that corresponds to an unfolded RNA conformation (Fig. 2 *D* and *E*). These results are not compatible with the RNA-sliding mechanism, which would be expected to yield intermediate FRET states, reminiscent of the mechanisms employed by positively charged disordered chaperones that enhance nucleic acid folding via local charge screening (45). Notably, NSP2-mediated RNA-unfolding is distinct from that of the other viral RNA chaperone avian reovirus σNS that was shown to destabilize RNA structures via multiple, dynamic interconverting intermediate states (4, 42). Thus, single-molecule biophysics data reveal that NSP2 is able to capture folded RNA stem-loop structure within a single RNA-binding groove. This results in general relaxation of RNA structure without fraying of the RNA stem termini, so that the RNA structure is sufficiently destabilized that it adopts a completely open conformation. Since NSP2 preferentially binds unstructured RNA (4), it forms a stable RNP complex with an unstructured RNA until intermolecular annealing is achieved. By binding to multiple RNAs concurrently via surface-exposed grooves (Fig. 7*A*, cyan) (4, 7, 19), NSP2 octamers act as matchmakers of complementary sequences, promoting intermolecular RNA–RNA interactions. Our single-molecule fluorescence and binding kinetics experiments also indicate that removal of the CTR does not perturb RNA binding but slows RNA release (∼3.2-fold increase in k_off_). Moreover, CTR removal results in a ∼2.4-fold increase in the RNA-unwinding activity of NSP2-∆C as well as an approximately twofold decrease in its RNA-annealing activity. Additionally, smFRET data reveal that binding to NSP2-∆C energetically favors low-FRET (unfolded) RNA conformations, resulting in remodelling of structured RNAs (Fig. 2*D*). The resulting increased stability of NSP2-∆C–RNA complexes precludes efficient RNA annealing, yielding kinetically trapped RNP complex intermediates (Fig. 7 *B* and *C*). We propose that conserved acidic patches within the ampholytic CTR (Fig. 7*A*, red) accelerate RNA displacement from NSP2 via charge repulsion, thus enabling RNA chaperone recycling and duplex release. Removal of the ampholytic CTRs in NSP2 variants derived from two distinct RV viruses (strains SA11 and RF) has a similar outcome on RNA chaperone activity in vitro (*SI Appendix*, Fig. S3), suggesting a conserved role of the CTR in NSP2 function. This model is further supported by our observation that removal of the unstructured region downstream of the ampholytic CTR (Fig. 1*A*) does not alter the RNA-unwinding activity of NSP2 (*SI Appendix*, Fig. S4). Indeed, this partial truncation has been previously shown to support viral replication (10). Excitingly, we have exploited recent technical advances in reverse genetics of RVs to directly demonstrate the pivotal role of these conserved acidic residues in RV replication (Fig. 6). Moreover, while a single NSP2 octamer can efficiently achieve RNA stem-loop unwinding at subnanomolar concentration, we have previously shown that intermolecular strand-annealing requires a stoichiometric excess of NSP2 octamers (19). Thus, it is likely that intermolecular annealing takes place between RNAs presented by multiple NSP2 octamers. However, given the current available data, we cannot rule out the possibility of intermolecular annealing within the same RNA-binding groove or via protruding single-stranded sections.

The described principle of CTR-assisted RNA dissociation from NSP2 is strikingly similar to that of the bacterial RNA chaperone protein Hfq (46–48). Hfq possesses an unstructured C-terminal domain (CTD) with an acidic tip that drives RNA displacement from the core of Hfq (49). Unlike the Hfq CTD, we do not observe competition between the CTR and RNA for NSP2_core_ binding (50, 51). It is important to note that it is possible that the CTR promotes RNA displacement only when tethered to NSP2_core_ (e.g., through increased effective concentration of the CTR in proximity to bound RNA). Nevertheless, the CTR modulates the kinetics and thermodynamics of NSP2–RNP complex formation to accomplish RNA chaperone recycling. We propose that this may represent a conserved mechanistic feature of multimeric RNA chaperones that capture RNA with high affinity and require auto-regulation to assist RNA dissociation in order to promote efficient matchmaking.

## Materials and Methods

### Proteins and RNAs.

NSP2 and NSP2-∆C (RVA strains SA11 and RF) were expressed and purified as described previously (19). RNAs used in this study are listed in *SI Appendix*, Table S2. RNA sequences S5, S6, and S11 were produced and labeled using previously described in vitro transcription and labeling protocols (19). Unstructured 20mer (labeled and unlabeled), unstructured 10mer (labeled), and biotinylated unstructured 10mer RNAs were purchased from Integrated DNA Technologies. Dual-labeled (*SI Appendix*, Table S2) stem-loop RNA was purchased from IBA Life Sciences.

### Negative-Stain EM and Data Processing.

For negative-stain grid preparation, 4 µL of sample (at various concentrations ranging from 100 to 500 nM) was incubated on glow-discharged (using PELCO easiGlow), carbon-coated Formvar 300-mesh Cu grid (Agar Scientific) for 90 s prior to blotting, stained twice with 20 µL 2% uranyl acetate (first stain immediately blotted, the second stain incubated for 20 s prior to blotting), and allowed to dry. Micrographs were collected on a FEI Tecnai 12 transmission electron microscope operated at 120 kV and equipped with a Gatan UltraScan 4000 charge-coupled device camera operated at a nominal magnification of 30,000 × (giving a 3.74 Å/pixel sampling on the object level). From 23 micrograph images taken with a nominal defocus of −3 µm, 14,740 particles were picked using template-based autopicking within Relion 3. Multiple rounds of two-dimensional and 3D classification resulted in the selection of a subset of 2,864 particles. These particles were used to determine an ∼22 Å resolution NSP2-∆C reconstruction with D4 symmetry applied.

### Cryo-EM and Data Processing.

NSP2 RNP complex was assembled by incubating NSP2 with 40mer RNA. A range of different NSP2: RNA ratios were tested, but ratios above 1 RNA: 2 NSP2 monomer resulted in immediate complex precipitation. A 1 RNA: 2 NSP2 molar ratio gives a stoichiometry of 1 RNA per binding site on NSP2, thereby ensuring saturation of NSP2 with RNA. To establish optimal cryo-EM grid conditions, grids with a range of NSP2 RNP concentrations were made. RNP complex was incubated at room temperature (25 °C) for 30 min prior to application to Quantifoil 1.2/1.3 holey carbon grids and vitrification. At 25 μM RNP complex (∼1 mg/mL), no particles were present in the ice; this was probably due to preferentially resting on the carbon instead of distributing evenly throughout the ice. This was ameliorated by a twofold increase in the RNP concentration (while maintaining a 1 RNA: 2 NSP2 monomer molar ratio), producing a suitable particle distribution within the ice. Further details on sample preparation, data acquisition and analysis are given in *SI Appendix*, *SI Methods*.

### Atomic Model Building.

An atomic model of NSP2 published by Jayaram et al. (52) (PDB 1L9V) was fit into the cryo-EM densities using ChimeraX (53) and subjected to automated flexible fitting and refinement using Namdinator (54). The Namdinator model was used for multiple iterative rounds of manual adjustment in Coot (55) and real-space refinement in Phenix (56). Models for NSP2 apoprotein and NSP2-RNP were validated using MolProbity (57) as implemented in Phenix.

### Protein Sequence Conservation and Surface Electrostatic Analyses.

Full-length NSP2-coding sequences of group A RVs were obtained from GenBank (*SI Appendix*, Table S4). Sequences of avian strains and of rearranged RNA segments of mammalian strains were excluded from the analysis. Protein sequence conservation and multiple-sequence alignment (MSA) was performed using the online ConSurf (58) server. Output from the ConSurf MSA was used to generate a sequence logo using the WebLogo server (59). Maps and models were visualized using ChimeraX (53), and the electrostatic surfaces were determined using the the Adaptive Poisson–Boltzmann Solver plugin (60).

### smFRET Measurements.

SmFRET measurements of freely diffusing, dual-labeled RNA stem-loops in the presence and absence of NSP2 were performed on a home-built confocal microscope, as described previously (4). Briefly, the samples were excited using pulsed interleaved excitation (61) at wavelengths of 532 and 640 nm (PicoTA, Toptica, and LDH-D-C-640, PicoQuant) with typical laser powers of 100 µW (measured before the 60× water immersion objective [Plan Apo IR 60×/ 1.27 WI Nikon, Düsseldorf, Germany]). Fluorescence signal was split between the green and red detection channels using a DualLine Z532/635 beamsplitter (AHF) and the emission spectra filtered using a Brightline 582/75 filter (Semrock) for green detection and HQ700/75 and ET700/75 filters (Chroma) for red detection. Measurements were performed in 8-well chamber slides (Nunc Lab-Tek, VWR) in a buffer composed of 1/3 phosphate-buffered saline (PBS, 45 mM NaCl, 3 mM phosphate, and 1 mM KCl), 1 mM Trolox to reduce photobleaching (62), and 0.01% (vol/vol) Tween20 to prevent sticking of the sample to the glass surface. The dual-labeled RNA stem-loop (Atto532/Atto647N-labeled) was diluted to 25 pM and incubated with 5 nM NSP2 (either full length or the ∆C mutant). Data were analyzed with the open-source software package PAM (63) using the same burst search parameters and correction factors as described in ref. 4. To determine species-selective fluorescence CFs, we defined two subpopulations based on the FRET efficiency *E*: the low-FRET population with *E* < 0.4 and the high-FRET population with *E* > 0.6. For each burst, the CF for acceptor photons after acceptor excitation was calculated including photons within a time window of 20 ms.

### SPR.

A Biacore 3000 was used to analyze the binding kinetics of NSP2 and NSP2-∆C to 5′biotinylated-10mer RNA (*SI Appendix*, Table S2). All experiments were performed in SPR buffer (150 mM NaCl, 25 mM Hepes, pH 7.5, and 0.1% Tween-20). RNAs were immobilized on a streptavidin (SA) sensor chip (GE Healthcare) with an analyte R_*max*_ of ∼20 resonance units (RU). Analyte measurements were performed at 25 °C and a flow rate of 40 µL/min. The chip surface was regenerated between protein injections with a 40 µL 0.05% sodium dodecyl sulfate (SDS) injection. Data were analyzed using BIAevaluation 3.1 software (GE Healthcare). The kinetic parameters were derived assuming a binding stoichiometry of 1:1.

### RNA Competition Assay.

A total 250 nM NSP2 and NSP2-∆C (RF) was preincubated with 10 nM 20mer AlexaFluor488-labeled RNA (*SI Appendix*, Table S2) in binding buffer (50 mM NaCl, 25 mM Hepes pH 7.5). Fluorescence anisotropy measurements were performed in the presence of various concentrations of unlabeled 20mer RNA in low-volume Greiner 384-well plates. Data were recorded at 25 °C in a PHERAstar Plus multidetection plate reader (BMG Labtech) equipped with a fluorescence polarization optical module (λ_ex_ = 485 nm; λ_em_ = 520 nm). The data were normalized and binding curves were fitted in Origin 9.0 using a Hill binding curve resulting in R^2^ values of 0.997 and 0.991 for NSP2 and NSP2-∆C respectively.

### CTR Peptide Competition Assay.

In order to maximize any potential competition between the CTR peptide and RNA, assays were performed under conditions that favored dissociation of NSP2-∆C from RNA. The binding assay was performed in PBS buffer (150 mM NaCl, 10 mM potassium phosphate, and 3 mM potassium chloride). A total 25 nM AlexaFluor488-labeled RNA was incubated with 20-fold excess NSP2-∆C (500 nM, RF strain). After 30 min at room temperature (∼25 °C), the CTR peptide was added in 20-fold excess of NSP2-∆C (i.e., 10 µM). To investigate direct CTR–RNA interactions, 25 nM 10mer RNA was also coincubated with 10 µM CTR peptide. Fluorescence anisotropy measurements of RNA alone, RNA-NSP2-∆C, RNA:NSP2-∆C:CTR, and RNA:CTR were performed in triplicate as described above for RNA competition assays.

### HDX-MS.

An automated HDX robot (LEAP Technologies, Ft Lauderdale, FL) coupled to an Acquity M-Class liquid chromatography and HDX manager (Waters, United Kingdom) was used for all HDX-MS experiments. Differential HDX-MS of NSP2 was performed using NSP2 (10 µM) or preincubated NSP2-RNP complexes (10 µM + 2 µM 20mer RNA, *SI Appendix*, Table S2). A total 30 µl of protein-containing solution was added to 135 μL deuterated buffer (10 mM potassium phosphate buffer pD 8.0, 82% D_2_O) and incubated at 4 °C for 0.5, 2, 30, or 120 min. After labeling, HDX was quenched by adding 100 μL of quench buffer (10 mM potassium phosphate, 2 M guanidine hydrochloride, and pH 2.2) to 50 μL of the labeling reaction. A total 50 μL of the quenched sample was passed through immobilized pepsin and aspergillopepsin columns (Affipro, Mratín, Czech Republic) connected in series (20 °C), and the peptides were trapped on a VanGuard Precolumn (Acquity UPLC BEH C18 [1.7 μm, 2.1 mm × 5 mm, Waters, United Kingdom]) for 3 min. The peptides were separated using a C18 column (75 μm × 150 mm, Waters, United Kingdom) by gradient elution of 0 to 40% (vol/vol) acetonitrile (0.1% vol/vol formic acid) in H_2_O (0.3% vol/vol formic acid) over 7 min at 40 μL/min^−1^. Peptides were detected using a Synapt G2Si mass spectrometer (Waters, United Kingdom). The mass spectrometer was operated in mobility-assisted data-independent analysis with the dynamic range extension enabled (HDMS^E^) mode were used to separate peptides prior to collision-induced dissociation (CID) fragmentation in the transfer cell. CID data were used for peptide identification, and uptake quantification was performed at the peptide level (as CID results in deuterium scrambling). Data were analyzed using PLGS (version 3.0.2) and DynamX (64) (version 3.0.0) software (Waters, United Kingdom). Restrictions for peptides in DynamX were as follows: minimum intensity = 1,000, minimum products per amino acid = 0.3, max sequence length = 25, max ppm error = 5, and file threshold = 3. The software Deuteros (65) was used to identify peptides with statistically significant increases/decreases in deuterium uptake (applying a 99% confidence interval) and to prepare Woods plots.

### Native MS.

NSP2 and its C-terminal truncation was extensively dialyzed into 200 mM ammonium acetate, pH 7.6 overnight at 4 °C. CTR peptide was diluted to a final concentration of 100 μM, and then further diluted to the appropriate concentration to achieve a 1:1 protein:peptide stoichiometric ratio. Nano Electro-spray ionisation (NanoESI)–IMS–MS spectra were acquired with an Orbitrap UHMR mass spectrometer (Thermo Fisher Scientific), operated as previously described (4). Data were processed using Xcalibur Qual Browser version 4.0.27.19 and UniDec version 2.7.1.

### UV Cross-linking MS with RBDmap.

A total 10 µM NSP2 was incubated with 5′-A_25_-S11 RNA in a final volume of 100 µL. NSP2–RNP complexes were incubated at room temperature for 30 min and applied to a single well of a 24-well plate. This 24-well plate was placed on an aluminum block cooled to 4 °C within a plastic container of ice and subjected to six rounds of UV irradiation (254 nm, 0.83 J/cm^−2^ per round) in a UVP CL-1000 UV Crosslinker (Scientifix). Crosslinked RNP complexes were digested by LysC (NEB, #P8109S) (500 ng per crosslinked RNP sample) overnight at room temperature. Enrichment and identification of cross-linked peptides were performed using the in vitro adaptation of the RBDmap protocol, as described in ref. 22. Data analysis was performed using the CrissCrosslinker R script, as described in ref. 22.

### Cells and Viruses.

MA104 cell line (embryonic African green monkey kidney cells, ATCC CRL-2378) and its derivatives stably expressing NSP5-EGFP (MA-NSP5-EGFP) and NSP5 (MA-NSP5) (31) were cultured in Dulbecco's Modified Eagle's Medium (DMEM) (Life Technologies) supplemented with 10% Fetal Bovine Serum (FBS) (Life Technologies). BHK-T7 cells (Baby hamster kidney stably expressing T7 RNA polymerase) were cultured in Glasgow medium supplemented with 5% FBS, 10% Tryptose Phosphate Broth (Sigma-Aldrich), 2% Non-Essential Amino Acid (Sigma-Aldrich), and 1% Glutamine.

### RV Reverse Genetics and Plasmids.

Recombinant simian RV strain SA11 NSP2 mutants were rescued using plasmids encoding the wild-type SA11 (G3P[2]) virus with modifications, as previously described (31). Further experimental details for are given in *SI Appendix*, *SI Methods*.

### smFISH.

MA104-NSP5-EGFP cells were seeded into Ibidi 8-well µ-slides and allowed to reach 90% confluency prior to the infection. Confluent cell monolayers were infected with trypsin-activated RV stocks, as described in ref. 66) at multiplicity of infection of 10. Cells were fixed at 6 to 8 hpi with 4% (vol/vol) paraformaldehyde in PBS for 10 min at room temperature, then rinsed twice with PBS, followed by immersion into 70% ethanol in RNase-free water. Further details for RNA hybridization and RNA detection are given in *SI Appendix*, *SI Methods*.

### Cell Transfections and Immunofluorescence Detection.

MA-NSP5 cells were seeded on glass coverslips (IBIDI) 24 h prior to transfection with pcDNA3-NSP2-AAA construct (*SI Appendix*, *SI Methods*) using Lipofectamine 3000 (Sigma-Aldrich), following the manufacturer’s instructions. The cells were maintained in DMEM supplemented with 10% FBS for 3 d, after which the cells were fixed with 4% (vol/vol) paraformaldehyde in PBS for 15 min at room temperature then rinsed twice with PBS and incubated for 10 min in a 0.1 M solution of glycine in PBS pH 7.4. Samples were then permeabilized with 0.2% Triton X-100 (PBS, vol/vol) for 10 min, rinsed with PBS, and incubated in PBS containing 3% molecular biology grade bovine serum albumin (BSA) (Sigma-Aldrich) for 1 h at room temperature. Cells were then incubated with guinea pig NSP5 antibodies (67) diluted 1:5,000 in PBS containing 3% molecular biology grade BSA, left at RT for 1 h, and washed three times with PBS containing 0.1% Tween-20, followed by coincubation with goat anti-guinea pig AlexaFluor550-labeled anti-guinea pig IgG (Invitrogen, cat.# A-21435), following the manufacturer’s instructions. Cells were washed three times with PBS prior to imaging.

### Image Data Acquisition.

Widefield imaging was carried out on an ONI Nanoimager S with an Olympus 100× super apochromatic oil immersion objective (NA1.4). Dye excitation was performed with an ONI laser illumination system at the wavelengths 405 nm (DAPI), 488 nm (eGFP), 561 nm (AlexaFluor550), and 640 nm (Atto647N), with laser intensities set to 10% (405 nm), 2% (488 nm), 5% (561 nm), and 7% (641 nm). Fluorescent signals were recorded with a sCMOS camera with a pixel size of 0.117 µm. Images were acquired over a field of view of the camera chip resulting in a total imaging region of 50 µm × 80 µm. Exposure times were adjusted accordingly to the signal intensity to avoid pixel saturation. Typical exposure times were 30 ms for all channels. Images were recorded consecutively for each channel, from the lowest to the highest energy excitation wavelength. All images were processed in ImageJ (68), and figures were assembled in CorelDraw2020.

## Supplementary Material

Supplementary File

## Data Availability

Sequencing data are available for rescued recombinant RVs (GenBank IDs: MW074066, MW074067, and MW074068). The following wwPDB accession codes have been assigned to the EM data: NSP2 (PDB 7PKO, EMD-13474), NSP2–RNP complex (7PKP, EMD-13476), and focused 3D class of the RNP (EMD-13475). All other data needed to evaluate the conclusions in the paper are present in the paper and/or *SI Appendix*.
