# Supplementary material for: Structural basis of rotavirus RNA chaperone displacement and RNA annealing
Source: Proc Natl Acad Sci U S A. 2021 Oct 6;118(41):e2100198118. doi: 10.1073/pnas.2100198118 (PMC8521686; doi:10.1073/pnas.2100198118)
Supplement: Supplementary File [file pnas.2100198118.sapp.pdf]

PNAS
www.pnas.org

**Supplementary Information for**

# Structural basis of rotavirus RNA chaperone displacement and RNA annealing

Jack P. K. Bravo, Kira Bartnik, Luca Venditti, Julia Acker, Emma H. Gail, Alice Colyer, Chen Davidovich, Don C Lamb, Roman Tuma, Antonio N. Calabrese, Alexander Borodavka*

*Corresponding author
Email: ab2677@cam.ac.uk

**This PDF file includes:**

SI Methods
Figures S1 to S8
Tables S1 to S3
Legends for Dataset S1
SI References

## SI Methods

### Cryo-EM data collection and processing

Cryo-EM was performed exclusively with Quantifoil R.1.2/1.3 holey carbon grids purchased from Quantifoil. All grids were glow discharged in air using GloQube glow discharge system (Quorum) immediately prior to use. All grids were prepared using a Vitribot IV (FEI) at 100% humidity and 4°C, with a blotting time of 6 seconds and a nominal blotting force of 6. Samples were flash-frozen in liquid nitrogen ($LN_2$)-cooled liquid ethane and immediately transferred to storage dewars under $LN_2$.

Vitrified samples were imaged at low temperature in-house (Astbury Biostructure Laboratory, University of Leeds), using Thermo Fisher Titan Krios microscopes equipped with either a Falcon III (NSP2 apoprotein) or a Gatan K2 (NSP2-RNP) detector. Data was collected with an acceleration voltage of 300 kV and a nominal magnification of 75,000x, resulting in pixel sizes of 1.065 Å (Falcon III) or 1.07 Å (K2). Data collection parameters are described in **Table S3**.

Image processing was carried out using the Relion 3 pipeline (1). Movie drift-correction was performed using MOTIONCOR2 (2), and the contrast transfer function of each movie was determined using gCTF (3). Initial particle autopicking of a subset of 5 – 10 randomly chosen micrographs was performed with the Laplacian-of-Gaussian (LoG) tool within the Autopicking module of Relion3. Particles were extracted and subjected to initial 2D classification in order to identify particles and assess autopicking success. Following this, the entire dataset was picked using LoG methods, extracted using 256 pixel box size and binned four times (effective box size 64 pixels) and subjected to 2D classification with fast subsets in order to remove false-positive particles that had been erroneously picked. Next, a more rigorous 2D classification was performed (without fast subsets). Particles originating from 2D classes with secondary structural features were selected and used to generate an initial model. Following multiple rounds of 3D and 2D classification, suitable particles were selected for 3D auto-refinement and various symmetry parameters were applied. Following refinement, per-particle CTF and Bayesian Polishing were performed in Relion 3, and 'shiny' particles were re-refined. Post-processing was performed with a soft mask of 15 pixels and the B-factor estimated automatically in Relion 3.

After particle polishing, the NSP2 apoprotein and the NSP2-RNP complex were subjected to further 3D classification into three classes without particle orientations. This yielded three similarly-sized subsets of near-identical particles for the NSP2 apoprotein whose resolution did not improve upon the original, larger dataset following 3D auto-refinement and post-processing. For the NSP2-RNP complex, this gave a single class with 86% of particles, and two other classes with 6% and 8% of the particles. The class with 6% of input particles had well-defined protein and RNA densities and was used for 3D auto-refinement and post-processing. This improved the map resolution from 3.5 Å to 3.4 Å with C4 symmetry. A D4 symmetry reconstruction further increased the map resolution from 3.5 Å to 3.1 Å.

For the NSP2 RNP complex, symmetry expansion was performed on a subset of the 635,599 particles used for a C4 symmetry reconstruction using the *relion_particle_symmetry_expand* command, generating four symmetry-related orientations for each particle. A mask covering a single basic groove-face of NSP2 was

made using the volume eraser tool in UCSF ChimeraX (4) and Relion 3, with a soft edge of 15 pixels (**Fig. S2**). The symmetry-expanded dataset was then subjected to focussed classification into 10 classes using this mask without particle orientations. Suitable classes (four classes containing >99% of input particles) were selected, and manually examined for putative RNA density. The subset of particles with the strongest RNA density feature were reconstructed without a mask, and subjected to masking (with the mask corresponding to the entire NSP2 octamer rather than a single face) and post-processing as described for reconstructions with D4 symmetry imposed. Sharpened asymmetric and D4 symmetry maps were aligned using the Fit-In-Map tool within UCSF Chimera and had a correlation of 0.9663.

**Rotavirus reverse genetics and plasmids**

Plasmids $pT_7$-VP1-SA11, $pT_7$-VP2-SA11, $pT_7$-VP3-SA11, $pT_7$-VP4-SA11, $pT_7$-VP6-SA11, $pT_7$-VP7-SA11, $pT_7$-NSP1-SA11, $pT_7$-NSP2-SA11, $pT_7$-NSP3-SA11, $pT_7$-NSP4-SA11, and $pT_7$-NSP5-SA11 (*11*) were used for recombinant virus rescue in reverse genetics experiments. $pT_7$-NSP2 plasmid variants (NSP2-EED, NSP2-AAA, and NSP2-6xHis) carrying mutations in gs8 were generated using Q5 site-directed mutagenesis (NEB), using mutagenesis primers (Table S2). pcDNA3-NSP2-AAA plasmid was generated from pcDNA3-NSP2 using Q5 site-directed mutagenesis (Table S2). To rescue recombinant RV strain SA11, monolayers of BHK-T7 cells ($4 \times 10^5$) cultured in 12-well plates were co-transfected using 2.5 µL of TransIT-LT1 transfection reagent (Mirus) per microgram of DNA plasmid. Each mixture comprised 0.8 µg of SA11 rescue plasmids: $pT_7$-VP1, $pT_7$-VP2, $pT_7$-VP3, $pT_7$-VP4, $pT_7$-VP6, $pT_7$-VP7, $pT_7$-NSP1, $pT_7$-NSP3, $pT_7$-NSP4, and 2.4 µg of $pT_7$-NSP2 and $pT_7$-NSP5 (*11*, *63*). Additional 0.8 µg of pcDNA3-NSP2 and 0.8 µg of pcDNA3-NSP5, encoding NSP2 and NSP5 proteins, were also co-transfected to increase the virus rescue efficiencies. At 24 h post-transfection, MA104 cells ($5 \times 10^4$ cells) were added to the transfected cells and co-cultured for 72 hours in FBS-free medium supplemented with trypsin (0.5 µg/mL, Sigma Aldrich). After incubation, transfected cells were lysed by repeated freeze-thawing and 0.2 ml of the lysate was transferred to a fresh MA104 cell monolayer. After adsorption at 37°C for 1 hour, followed by a 5 min wash with PBS, cells were further cultured for 4 days in FBS-free DMEM supplemented with 0.5 µg/mL trypsin until a clear cytopathic effect (CPE) was visible. For AAA mutant, where CPE was not observed after 4 days of incubation, cells were harvested and lysed by freeze-thawing, and the clarified lysates were used for two additional blind virus passages, during which RNA samples were extracted from these lysates for further verification of viral replication by RT-PCR using gs8-specific primers (Table S2). These recombinant viruses were verified by DNA sequencing of the gs8-NSP2 RT-PCR products (GenBank IDs: MW074066, MW074067, MW074067).

**Single-molecule (sm) RNA FISH**

PFA-fixed RV-infected cells stored in ethanol at +4°C for at least 12 hours prior to hybridization were re-hydrated for 5 min in a pre-hybridization buffer (300 mM NaCl, 30 mM trisodium citrate, pH 7.0 in nuclease-free water, 10 % v/v Hi-Di formamide (Thermo Scientific), supplemented with 2 mM vanadyl ribonucleoside complex). Re-hydrated samples were hybridized with an equimolar mixture of ATTO647N-dye labelled DNA probes specific to gene segment 5 of RVA, strain SA11, 62.5 nM final concentration, in a total volume of 200 *µ*l of the hybridization buffer (Stellaris RNA FISH hybridization buffer, Biosearch Technologies, supplemented with 10% v/v Hi-Di formamide). After 4 hours of incubation at 37°C in a humidified chamber, samples were briefly rinsed with the wash

buffer (300 mM NaCl, 30 mM trisodium citrate, pH 7.0, 10 % v/v formamide in nuclease-free water, after which a fresh aliquot of 300 μl of the wash buffer was applied to each well and incubated twice at 37°C for 30 min. Sequences of the oligonucleotide RNA FISH probes specific for gs5 (NSP1, strain SA11, GenBank ID: KF729657.1) were used (7). These were generated using the Stellaris RNA FISH probe designer (https://www.biosearchtech.com/stellaris-designer), using each gene-specific ORF and level 2 masking.

**NSP2 (Bovine rotavirus strain RF) threading and CTR peptide modelling**

Alignment of NSP2(SA11) and NSP2(RF) sequences was performed using T-Coffee (8). Threading of the NSP2(RF) sequence (based on the SA11 structure, PDB 1L9V) was performed using I-TASSER (9). CTR peptide modelling was carried out using trRosetta (10) and PEP-FOLD3 (11, 12).

**Circular dichroism (CD)**

CD experiments were performed in a Chirascan plus spectrometer (Applied Photophysics). Samples were prepared by dissolving the CTR peptide in 10 mM phosphate buffer pH 7.4, 200 mM sodium fluoride. Spectra were recorded over a wavelength range of 190–260 nm with a bandwidth of 1 nm, step size of 1 nm and a path length of 1 mm. An average of three scans were used for the final spectra.

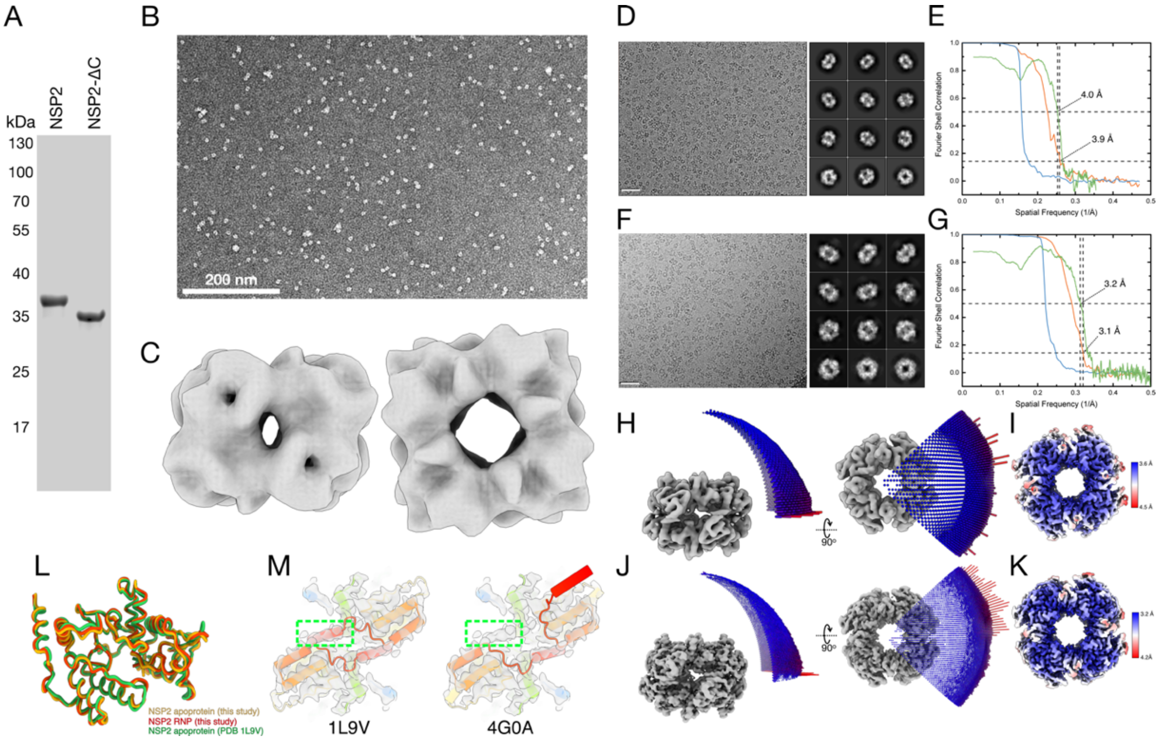


**Fig. S1. NSP2–ΔC variant assembles into octamers.**

**A:** SDS-PAGE of purified NSP2 and NSP2-ΔC. **B:** Representative negative stain EM micrograph of NSP2-ΔC **C:** 3D reconstruction of NSP2-ΔC. **D**: A representative cryo-EM micrograph of the NSP2 apoprotein and corresponding 2D class averages. **E:** The Fourier shell correlation (FSC) curves for the overall NSP2 apoprotein map (orange) and phase randomized map (blue). The map has a final resolution of 3.9 Å. Model versus map FSC curve (green) indicates a model resolution of 4.0 Å. **F:** A representative cryo-EM micrograph of the NSP2 RNP complex and corresponding 2D class averages. **G:** The Fourier shell correlation (FSC) curves for the overall NSP2-RNP complex map (orange) and phase randomized map (blue). Map has a final resolution of 3.2 Å. Model versus map FSC curve (green) indicates a model resolution of 3.1 Å. **H & J:** Euler angle distribution of particles corresponding to NSP2 apoprotein and NSP2-RNP reconstructions. The cylinder height and color represent the number of particles (blue to red – low to high). A D4 symmetry has been applied for both the NSP2 apoprotein and the NSP2-RNP complex reconstructions. **I & K:** NSP2 apoprotein (F) and NSP2-RNP complex (H) reconstructions colored by local resolution as calculated by Relion. **L:** Aligned models of the NSP2 apoprotein and the NSP2-RNP complex built from reconstructions presented in this study (yellow and red, respectively), and a previous crystal structure of the NSP2 apoprotein (PDB ID 1L9V) (green). Alignment of the apoprotein and RNP models from this study to 1L9V had an RMSD of 1.081 Å and 0.772 Å, respectively. The two models from this study had an RMSD of 0.742 Å. **M:** Fitting of previous NSP2 crystal structures into the NSP2 apoprotein cryo-EM density map. The “open” NSP2 conformation (PDB 4G0A) has the CTR flipped outwards, while the “closed” conformation (PDB 1L9V) has the CTR making contacts with the $NSP2_{core}$. The open conformation is not represented by the cryo-EM map, as the CTH density is clearly visible and localized to the $NSP2_{core}$. The green box highlights the cryo-EM density corresponding to the C-terminus.

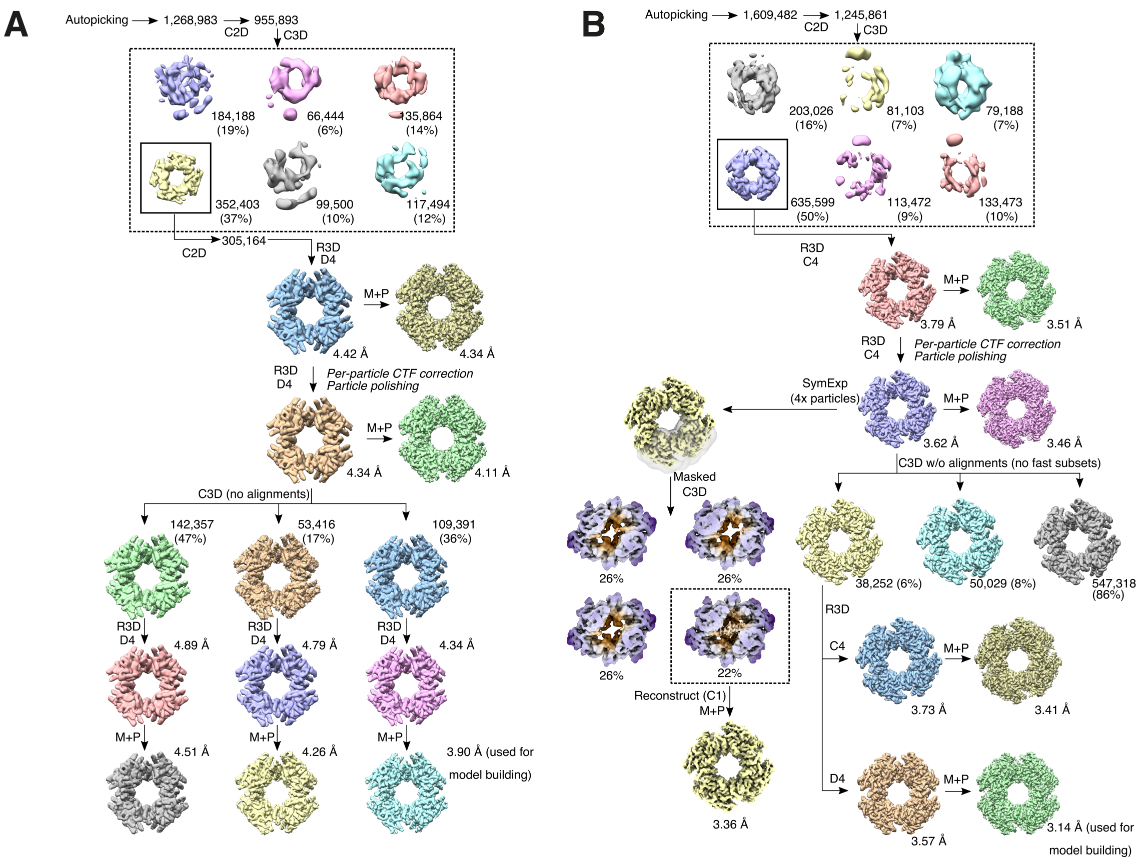


**Fig. S2. Cryo-EM structure determination of the NSP2 apoprotein and the NSP2-RNP complex.**

**A**: The image processing workflow used to determine the 3D reconstruction of the NSP2 apoprotein. Initial rounds of 2D classification (C2D) and 3D classification (C3D) were performed using Fast Subsets and image alignments with 25 iterations. The highest quality 3D class average was subjected to further 2D classification without fast subsets, and used for 3D refinement (R3D) with D4 symmetry imposed. After per-particle CTF correction and particle polishing, further 3D classification was performed without fast subsets, and without performing image alignment. 3D reconstructions were ultimately subjected to masking and post-processing (M + P). **B**: The image processing scheme used to determine the 3D reconstruction of NSP2 RNP. Initial 2D and 3D classifications were performed with fast subsets. Purple: Focused classification of NSP2-RNP demonstrate the existence of an RNA-binding groove with variable occupancy. Exemplary 3D class averages of NSP2-RNP with strong (boxed) and weak (all other) RNA density features.

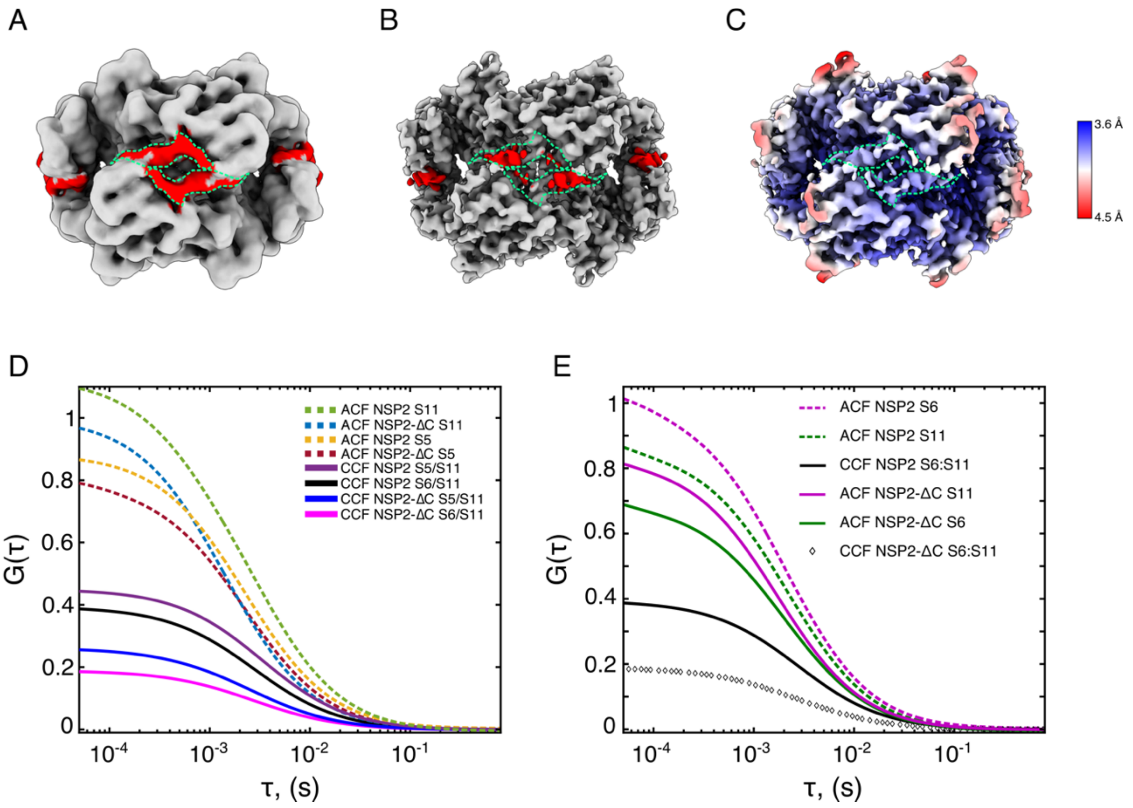


**Fig. S3. Role of the CTR in promoting RNA-RNA interactions.**
**A & B:** Unsharpened (A) and sharpened (B) NSP2 apoprotein cryo-EM density maps. Density corresponding to the CTR is shown in red (green outline). Linker density is diffuse in the sharpened map (B). **C:** Local resolution of NSP2 with the CTR outlined in green. **D:** Autocorrelation functions (ACFs) of rotavirus (RV) segments S5 and S11 in the presence of NSP2 and NSP2-ΔC (dashed lines). Cross-correlation functions (CCFs) (bold lines) of segments S5 and S11 in the presence of NSP2 (burgundy) and NSP2-ΔC (navy) and segments S6 and S11 in the presence of NSP2 (black) and NSP2-ΔC (magenta). **E:** ACFs and CCFs of S6 and S11 in the presence of NSP2 and NSP2-ΔC. Dashed, colored lines correspond to ACFs of WT NSP2 in complex with RV S6 and S11 RNAs, and continuous, colored lines correspond to ACFs of NSP2-ΔC in complex with RV S6 and S11 RNAs. Continuous black line and diamonds correspond to CCFs of both S6 and S11 in the presence of NSP2 and NSP2-ΔC, respectively.

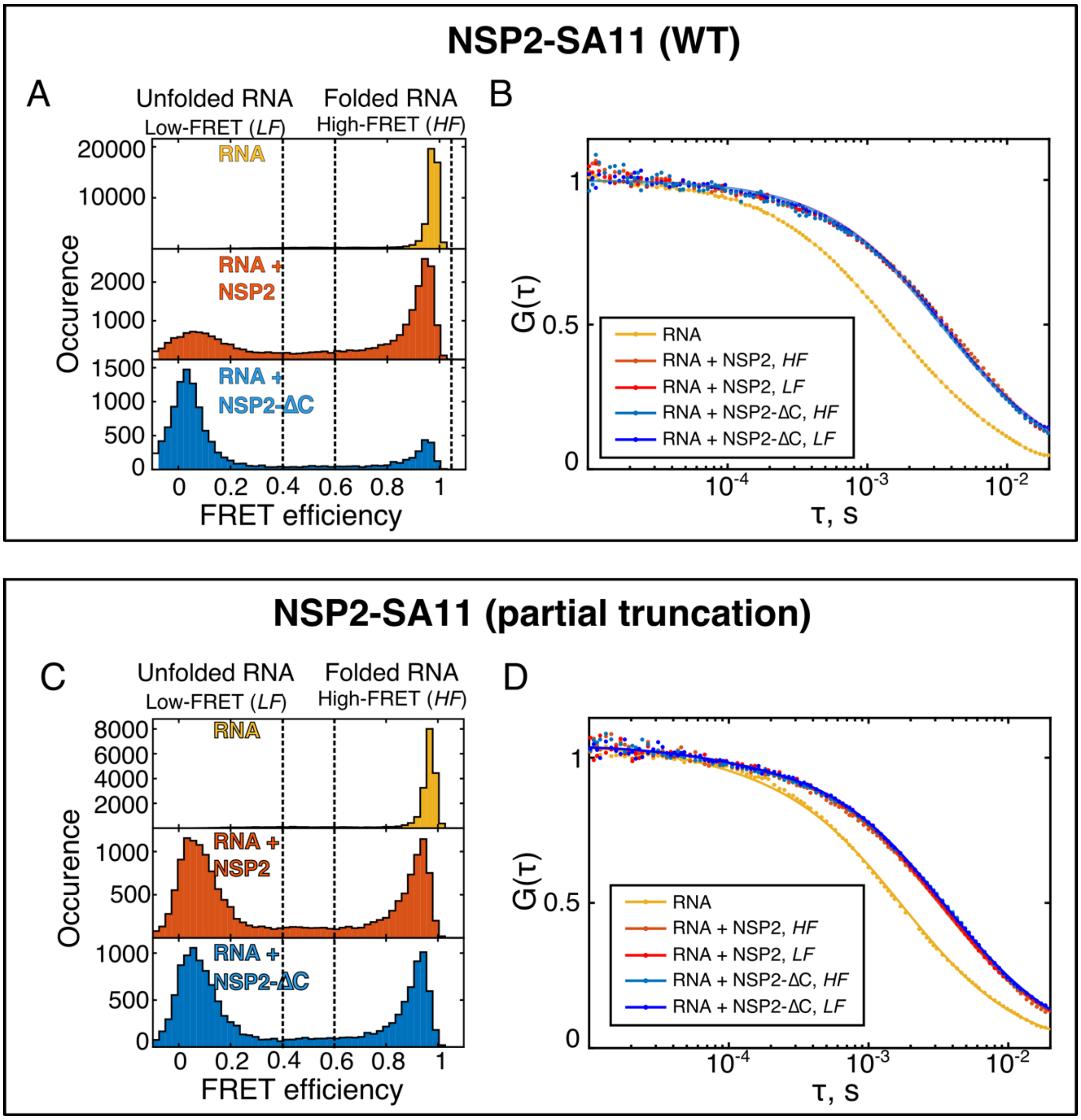


**Fig. S4. SmFRET and burstwise FCS analysis of an RNA stem-loop unwinding by NSP2 variants.**

**A & B:** SmFRET histograms (**A**) and corresponding burstwise FCS analysis (**B**) of the NSP2 RF strain and NSP2(RF)-ΔC. **C & D** smFRET histograms (**C**) and corresponding burstwise FCS analysis (**D**) of the NSP2 SA11 strain and a partially truncated NSP2 (lacking the unstructured residues 314-316). These residues are not essential for virus replication (13), and do not contribute to RNA unwinding.

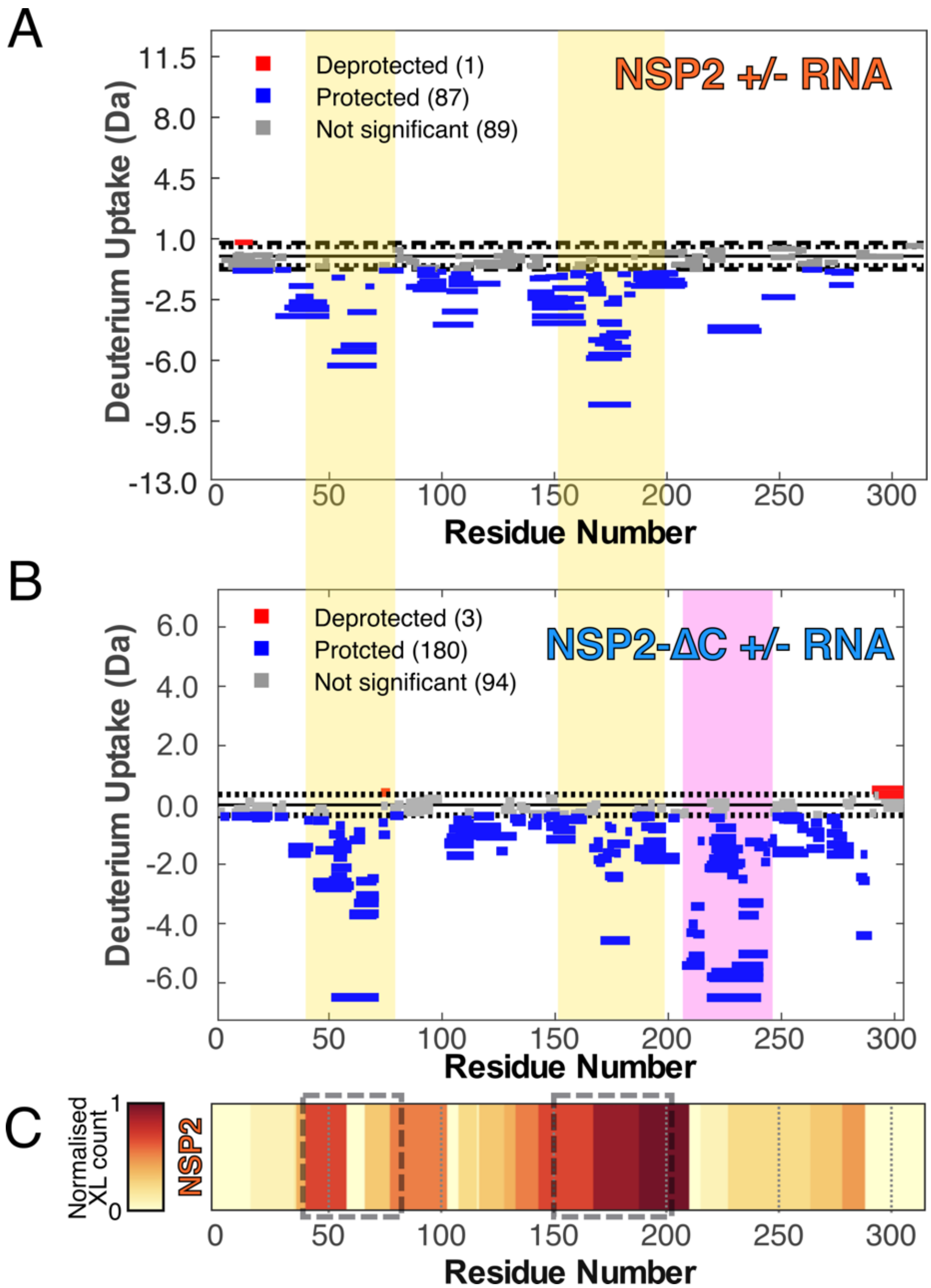


**Fig. S5. A & B:** Woods plots showing summed differences in deuterium uptake in NSP2 **(A)** and NSP2-ΔC **(B)** over all timepoints, comparing the apoproteins with their respective RNP complex. Regions corresponding to flexible loops located within the polar, equatorial groove of NSP2 are denoted by yellow shading. In NSP2-ΔC, the magenta box denotes residues that would be otherwise buried underneath the CTR. This includes R240. **C**: A heatmap of crosslinked peptides mapped onto the NSP2 sequence (plotted as counts per residue). Residues corresponding to flexible loops within the equatorial grove are denoted by grey boxes. These regions correspond to the yellow-shaded areas in the above Woods plots.

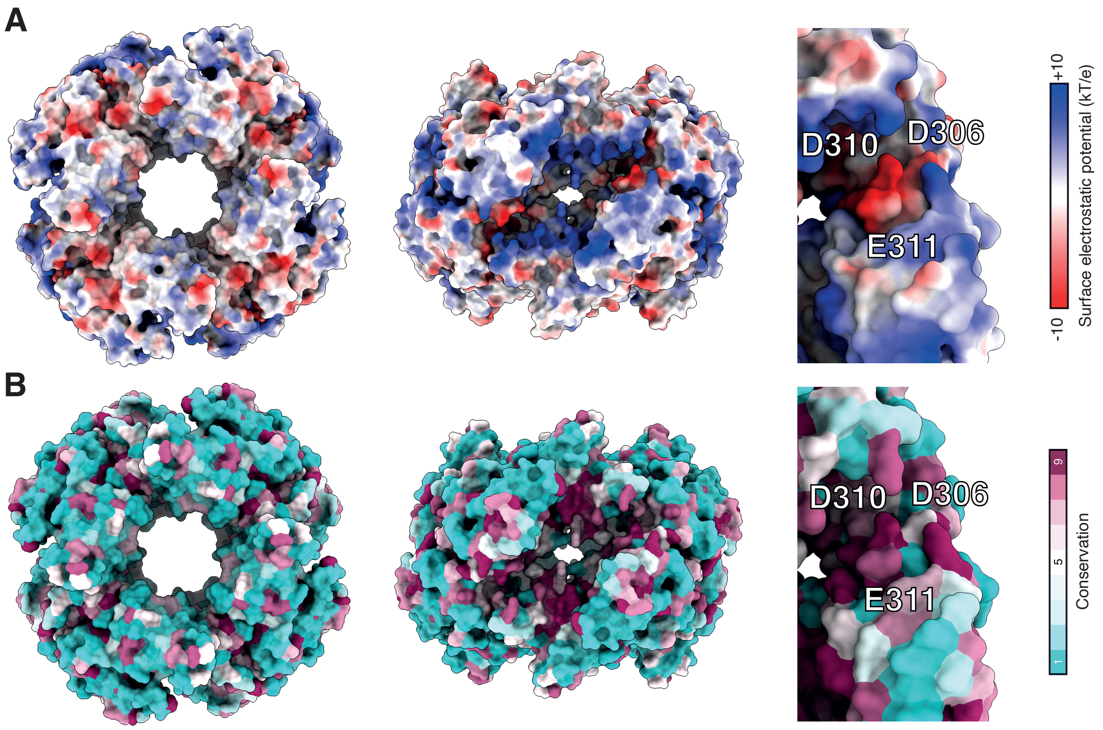


**Fig. S6.** Surface electrostatic potential (A), and conservation (B) analysis of NSP2 (See Materials and Methods for details). While the top view (left) shows no significant regions of high conservation, the side view (middle) reveals that the positively-charged groove responsible for RNA-binding is also highly conserved. Close-up view of the CTR acidic patch (right) shows that this region is also highly conserved. Conservation was calculated from the full-length NSP2 sequences (Rotavirus A, see Table S4) using ConSurf, as described in Materials and Methods.

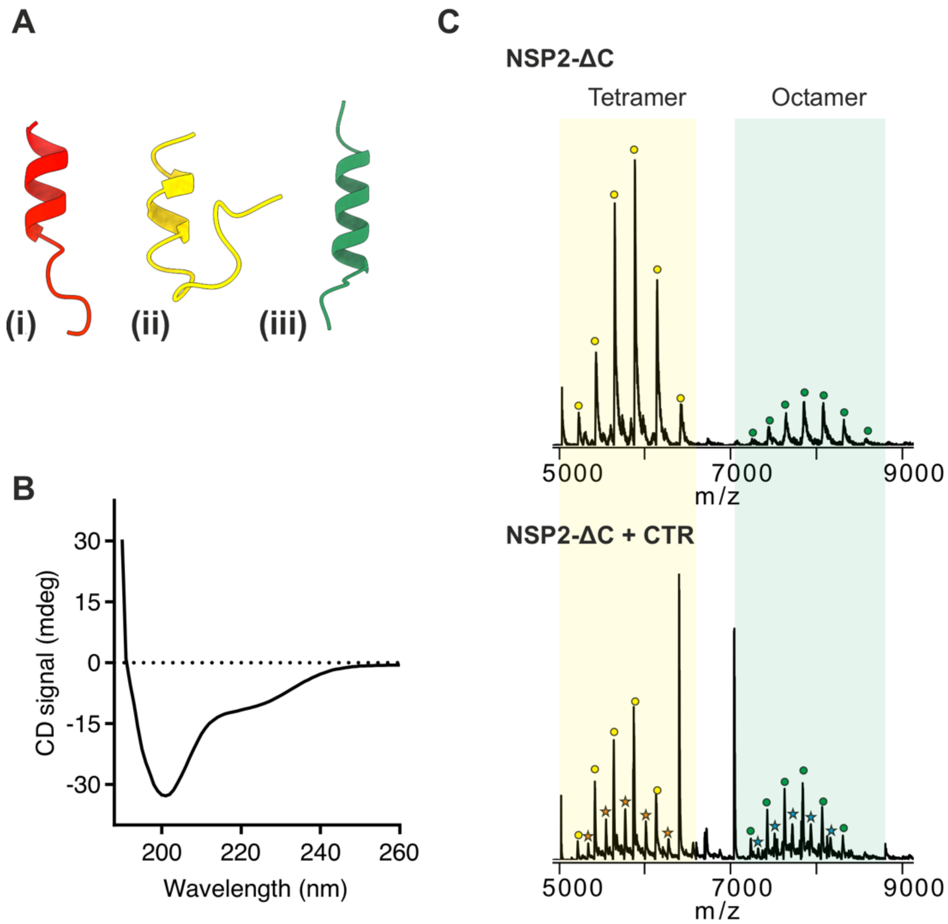


**Fig. S7. CTR peptide can directly bind to NSP2-ΔC octamer and retains its predicted conformation.**

A. Modelled structures of the CTR peptide. Our model (i), PEP-FOLD model (ii) (11, 12), and trRosetta model (iii) (10) were used to predict the 3D structure of the peptide. Calculated RMSD value for trRosetta model vs our model is 0.477Å, for PEP-FOLD model vs our model RMSD is 0.292Å, showing that predictions are in agreement with the structure of the CTR in the context of full-length NSP2. B. Circular Dichroism (CD) analysis of the isolated CTR peptide in solution, confirming that the solvated peptide assumes partially $\alpha$-helical conformation (ca. 15 % helicity), consistent with both the modelled CTR structure alone (see A), and the structural properties of this region in the context of full-length NSP2 (which is only partially helical and mostly disordered).

C. ESI-MS analysis of NSP2-ΔC alone (top panel) or NSP2-ΔC and CTR (dissolved in 150 mM ammonium acetate buffer, pH 7.5) incubated at 1:1 molar ratio (bottom panel) (5-20 $\mu$M range). Ions corresponding to tetrameric (yellow circles) and octameric (green circles) NSP2 species are observed, with dissociation of the octamer likely resulting from the weakened hydrophobic forces within the gas phase. Stoichiometric binding of the CTR peptide to NSP2-ΔC (orange stars, binding to tetramer and blue asterisk, binding to octamer) species confirms that the CTR peptide retains its ability to interact with NSP2-ΔC octamers in *trans*.

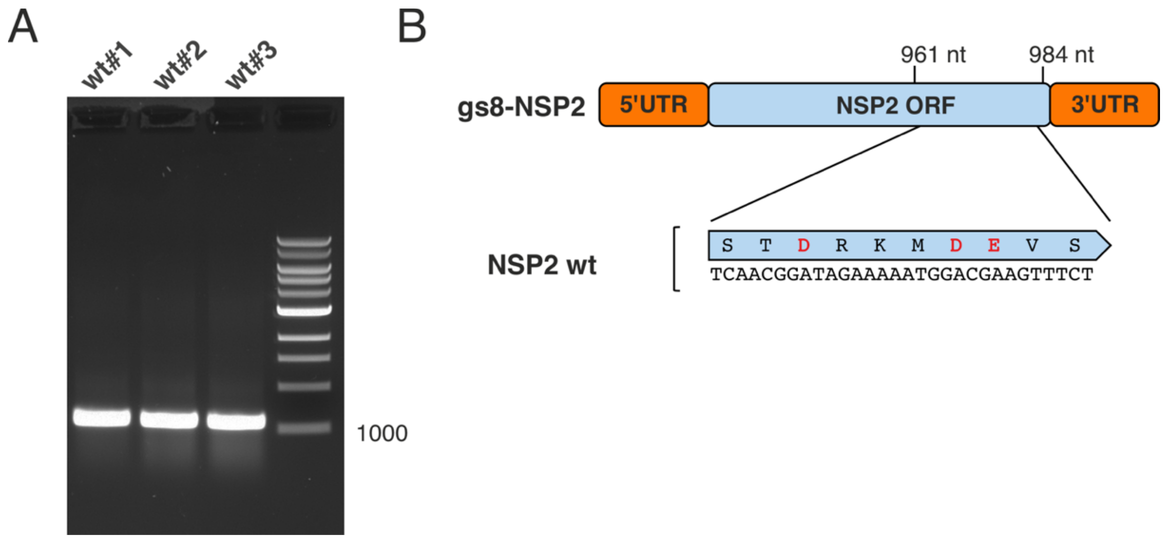


**Fig. S8. Rotavirus reverse genetics and wild-type NSP2 rescue.** A. RT-PCR results of the RNA extracted from MA104 cells infected with lysates from reverse genetics experiments employed to rescue wild-type (WT) SA11 virus (full-length NSP2). After 24 hpi, a cytopathic effect (CPE) was observed. Cells were lysed and RNA was extracted using QIAGEN RNEasy kit following the manufacturer's protocol. For each experiment, three independent attempts were made to rescue the wild-type virus and the mutants. Total RNA was reverse-transcribed and amplified using gs8-specific primers (Materials and Methods), prior to being sequenced. B. Representative sequencing result for a cDNA sample after amplification of the rescued WT NSP2 virus. Critical for viral replication acidic residues D306, D310, E311 are highlighted in red.

**Table S1. Binding kinetics of NSP2 and NSP2-ΔC as measured by SPR.** The association ($K_{on}$) and dissociation ($K_{off}$) rate constants obtained by SPR are given below.

<table>
<tr><th>RNA used</th><th>Sample</th><th>[NSP2] (nM)</th><th>$K_{on}$ ($M^{-1}s^{-1}$)</th><th>$K_{off}$ ($s^{-1}$)</th><th>$K_D$ (pM)</th><th>$\chi^2$</th><th>ΔOFF**</th></tr>
<tr><td rowspan="6">10mer RNA</td><td rowspan="3">NSP2 WT</td><td>6.25</td><td>$1.37 \times 10^{6}$</td><td>$9.04 \times 10^{-4}$</td><td>6580</td><td>1.742</td><td rowspan="3">N/A</td></tr>
<tr><td>12.5</td><td>$6.71 \times 10^{5}$</td><td>$8.58 \times 10^{-4}$</td><td>1280</td><td>15.23</td></tr>
<tr><td>25</td><td>$5.12 \times 10^{5}$</td><td>$7.20 \times 10^{-4}$</td><td>1410</td><td>128.7</td></tr>
<tr><td rowspan="3">NSP2 ΔC</td><td>6.25</td><td>$8.24 \times 10^{5}$</td><td>$2.92 \times 10^{-4}$</td><td>354</td><td>15.85</td><td>3.10</td></tr>
<tr><td>12.5</td><td>$4.03 \times 10^{5}$</td><td>$2.40 \times 10^{-4}$</td><td>596</td><td>30.43</td><td>3.58</td></tr>
<tr><td>25</td><td>$4.95 \times 10^{5}$</td><td>$2.46 \times 10^{-4}$</td><td>497</td><td>72.05</td><td>2.93</td></tr>
</table>

**Table S2. Sequences of RNAs used in the study**

| RNA substrate | Sequence (5' – 3') |
|---|---|
| **Unstructured 20mer (unlabeled) (used for RNA competition assay Fig. 7A)** | CUUUUCAAGACAUGCAACAA |
| **Unstructured 20mer (labelled) (used for RNA competition assay Fig. 7A)** | AF488-CUUUUCAAGACAUGCAACAA |
| **Unstructured 10mer (labelled) (used for peptide competition assay Fig. 7B)** | AF488-CUUCUUUCGA |
| **Biotinylated unstructured 10mer (SPR, Fig. 6)** | Biotin-CUUCUUUCGA |
| **Dual-labelled stem loop (smFRET, Fig. 3)** | ATTO532-AAAUCUUUGCAAACUAUCCAAUCCAUGCAAAGAUAA-ATTO647N |
| **40mer RNA (cryo-EM)** | CUUUUCAAGACAUGCAACAACUUUUCAAGACAUGCAACAA |
| **RV S5 (GenBank ID)** | KF729657.1 |
| **RV S6 (GenBank ID)** | KF729692.1 |
| **RV S11 (GenBank ID)** | KF729697.1 |
| **Q5 SDM primers – NSP2-6xHis** | FOR: ATGATGATGAACGCCAACTTGAGAAAC<br>REV: CACCACCACTAATTCGCTATCAATTTGAG |
| **Q5 SDM primers – NSP2-DEE** | FOR: ATGGAAGACGTTTCTCAAGTTGGCGTTTAATTC<br>REV: TTTTCTTTCCGTTGACAGCCCTTTAAATG |
| **Q5 SDM primers – NSP2-AAA** | FOR: ATGGCCGCGGTTTCTCAAGTTGGCGTTTAATTC<br>REV: TTTTCTTGCCGTTGACAGCCCTTTAAATG |
| **gs8-NSP2 sequencing/RT-PCR** | FOR: GAAATCAACACTGATTGCTATTG<br>REV: CCATCATCATCCTCAAATTG |

## Table S3. Cryo-EM data collection and refinement statistic

| | NSP2 apoprotein | NSP2 RNP |
|---|---|---|
| **Data collection and processing** | | |
| Nominal Magnification | 75,000 | |
| Voltage (kV) | 300 kV | |
| Defocus range (μm) | -1.2 to -2.4 | -1.5 to -3.5 |
| Detector | Falcon III | Gatan K2 |
| Pixel size (Å) | 1.065 | 1.07 |
| Electron exposure ($e^-/Å^2$) | 110 | 55 |
| Symmetry imposed | D4 | |
| Particles in first 3D classification | 955,893 | 1,245,861 |
| Final particle number | 109,391 | 38,252 |
| Nominal map resolution (Å) | 3.90 | 3.14 |
| B-factor* | -239.2 | -100.8 |
| FSC threshold | 0.143 | |
| Map resolution range (Å) | 3.64 – 4.46 | 3.22 – 4.19 |
| **Refinement, model composition and validation** | | |
| Initial model used | PDB 1L9V | |
| Model resolution (Å) | 4.0 | 3.2 |
| FSC threshold | 0.5 | |
| Non-hydrogen atoms | 20368 | 20368 |
| RMSD bonds (Å) | 0.004 | 0.008 |
| RMSD angles (°) | 0.651 | 0.810 |
| ***Ramachandran*** | | |
| Favoured (%) | 92.52 | 93.01 |
| Allowed (%) | 7.48 | 6.99 |
| Outliers (%) | 0.00 | 0.00 |
| Rotamer outliers (%) | 0.00 | 0.00 |
| Clash score | 10.77 | 7.13 |
| Molprobity score | 2.02 | 1.84 |
| ***Model-to-map fit*** | | |
| Cross-correlation coefficient (mask) | 0.82 | 0.83 |
| Cross-correlation coefficient (volume) | 0.81 | 0.81 |
| Main-chain | 0.81 | 0.83 |
| Sidechain | 0.78 | 0.81 |

**Table S4. Rotavirus (group A) strains and sequences used for NSP2 sequence conservation analysis.**

| Accession # | Host | Strain | Accession # | Host | Strain |
|---|---|---|---|---|---|
| J02420 | Bovine | UK | EF990710 | Human | B3458 |
| EF554133 | Human | PA169 | EU169872 | Porcine | SB1A |
| EF990700 | Bovine | WC3 | DQ146689 | Human | N26 |
| EF554144 | Human | 1110527 | DQ146678 | Human | Matlab13 |
| EF554122 | Human | B10925 | EF560709 | Human | Dhaka6 |
| EF554089 | Human | B1711 | DQ492676 | Human | Dhaka16 |
| EF554155 | Porcine | OVR762 | DQ146659 | Human | Dhaka25 |
| AF506018 | Human | NR1 | DQ494402 | Bovine | KJ75 |
| DQ005118 | Human | DRC86 | DQ494401 | Bovine | KJ44 |
| DQ005107 | Human | DRC88 | X57944 | Human | SG2 |
| L04530 | Human | NCDV | X06722 | Porcine | OSU |
| J02353 | Simian | SA11 | DQ146645 | Bovine | B4633 |
| DQ490541 | Human | RV161 | AF506293 | Bovine | RMC321 |
| X94562 | Human | IS2 | AF506014 | Bovine | RMC100 |
| DQ490558 | Human | RV176 | DQ146667 | Bovine | Dhaka12 |
| AY787648 | Human | TBChen | AB022770 | Bovine | KU |
| Z21640 | Bovine | RF | L04534 | Bovine | WA |
| EF554111 | Human | Hun5 | EF990696 | Porcine | A253 |
| EF554100 | Human | MG6 | EF990692 | Porcine | A411 |
| AY740734 | Human | B4106 | EF990688 | Porcine | A131 |
| DQ205227 | Lapine | 3096 | L04532 | Simian | SA11R |
| L04529 | Human | DS1 | DQ490534 | Human | AU1 |
| EF990704 | Bovine | BRV033 | L04531 | Simian | SA11P |
| DQ146696 | Human | L26 | DQ146703 | Human | T152 |

## SI References


1. J. Zivanov, *et al.*, RELION-3: new tools for automated high-resolution cryo-EM structure determination. *Elife*, 421123 (2018).
2. S. Q. Zheng, *et al.*, MotionCor2: Anisotropic correction of beam-induced motion for improved cryo-electron microscopy. *Nat. Methods* **14**, 331–332 (2017).
3. K. Zhang, Gctf: Real-time CTF determination and correction. *J. Struct. Biol.* **193**, 1–12 (2016).
4. T. D. Goddard, *et al.*, UCSF ChimeraX: Meeting modern challenges in visualization and analysis. *Protein Sci.* **27**, 14–25 (2018).
5. Y. Kanai, *et al.*, Entirely plasmid-based reverse genetics system for rotaviruses. *Proc. Natl. Acad. Sci.* **9**, 2349–2354 (2017).
6. G. Papa, *et al.*, Recombinant rotaviruses rescued by reverse genetics reveal the role of NSP5 hyperphosphorylation in the assembly of viral factories. *J. Virol.* **94**, 1–23 (2019).
7. S. Strauss, *et al.*, Principles of RNA recruitment to viral ribonucleoprotein condensates in a segmented dsRNA virus. *bioRxiv*, 2021.03.22.435476 (2021).
8. P. Di Tommaso, *et al.*, T-Coffee: A web server for the multiple sequence alignment of protein and RNA sequences using structural information and homology extension. *Nucleic Acids Res.* **39**, 13–17 (2011).
9. A. Roy, A. Kucukural, Y. Zhang, I-TASSER: A unified platform for automated protein structure and function prediction. *Nat. Protoc.* **5**, 725–738 (2010).
10. J. Yang, *et al.*, Improved protein structure prediction using predicted interresidue orientations. *Proc. Natl. Acad. Sci.* **117**, 1496 LP – 1503 (2020).
11. P. Thévenet, *et al.*, PEP-FOLD: an updated de novo structure prediction server for both linear and disulfide bonded cyclic peptides. *Nucleic Acids Res.* **40**, W288–W293 (2012).
12. A. Lamiable, *et al.*, PEP-FOLD3: faster de novo structure prediction for linear peptides in solution and in complex. *Nucleic Acids Res.* **44**, W449–W454 (2016).
13. Z. F. Taraporewala, *et al.*, Structure-function analysis of rotavirus NSP2 octamer by using a novel complementation system. *J. Virol.* **80**, 7984–94 (2006).
